# c-Jun inhibition mitigates chemotherapy-induced neurotoxicity in iPSC-derived sensory neurons

**DOI:** 10.1038/s41420-025-02847-5

**Published:** 2025-11-13

**Authors:** Lois Hew, Smilla K. Maierhof, Andranik Ivanov, Dieter Beule, Valeria Fernandez Vallone, Harald Stachelscheid, Silke Frahm, Narasimha Swamy Telugu, Matthias Endres, Wolfgang Boehmerle, Petra Huehnchen, Christian Schinke

**Affiliations:** 1 Charité – Universitätsmedizin Berlin, Corporate Member of Freie Universität Berlin and Humboldt Universität zu Berlin, Klinik und Hochschulambulanz für Neurologie, Berlin, Germany; 2 Charité – Universitätsmedizin Berlin, Corporate Member of Freie Universität Berlin and Humboldt-Universität zu Berlin, Einstein Center for Neurosciences Berlin, Berlin, Germany; 3 Berlin Institute of Health at Charité – Universitätsmedizin Berlin, Berlin, Germany; 4 Berlin Institute of Health at Charité – Universitätsmedizin Berlin, Core Unit Bioinformatics, Berlin, Germany; 5 Berlin Institute of Health at Charité – Universitätsmedizin Berlin, Core Unit Pluripotent Stem Cells and Organoids (CUSCO), Berlin, Germany; 6 Max Delbrück Center for Molecular Medicine in the Helmholtz Association (MDC), Technology Platform Pluripotent Stem Cells, Berlin, Germany; 7 Charité – Universitätsmedizin Berlin, Corporate Member of Freie Universität Berlin and Humboldt Universität zu Berlin, Center for Stroke Research Berlin (CSB), Berlin, Germany; 8 German Center for Neurodegenerative Diseases (DZNE), Partner Site Berlin, Berlin, Germany; 9 German Center for Cardiovascular Research (DZHK), Partner Site Berlin, Berlin, Germany; 10 German Center for Mental Health (DZPG), Partner Site Berlin, Berlin, Germany

**Keywords:** Mechanisms of disease, Peripheral nervous system, Preclinical research

## Abstract

Chemotherapy-induced peripheral neuropathy (CIPN) affects up to two-thirds of cancer patients undergoing cytotoxic chemotherapy. Here, we used human iPSC-derived sensory neurons (iPSC-DSN) to model CIPN in vitro. Administration of various chemotherapeutic agents (i.e., paclitaxel, vincristine, bortezomib and cisplatin) at clinically applicable concentrations resulted in reduced cell viability, axonal degeneration, electrophysiological dysfunction and increased levels of phosphorylated c-Jun in iPSC-DSN. Transcriptomic analyses revealed that the upregulation of c-Jun strongly correlated with the expression of genes of neuronal injury, apoptosis and inflammatory signatures. To test whether c-Jun plays a central role in the development of CIPN, we applied the small molecule inhibitor of the Jun N-terminal kinase, SP600125, to iPSC-DSN treated with neurotoxic chemotherapy. c-Jun inhibition prevented chemotherapy-induced neurotoxicity by preserving cell viability, axonal integrity and electrophysiological function of iPSC-DSN. These findings identify c-Jun as a key mediator of CIPN pathophysiology across multiple drug types and present preclinical evidence that c-Jun inhibition is an attractive therapeutic target to prevent CIPN.

## Introduction

Chemotherapy-induced peripheral neuropathy (CIPN) is an adverse event frequently incurred by cancer patients undergoing cytotoxic chemotherapy. Up to two-thirds of these patients develop CIPN and experience debilitating symptoms which may include numbness, tingling paresthesia, allodynia and pain, among other predominantly sensory deficits [1]. Such problems severely diminish the patients’ quality of life and chronic symptoms lasting over 20 years have been reported [1]. Furthermore, cancer treatment is often changed or terminated early when more severe symptoms of neuropathy arise [1, 2]. Unfortunately, many first-line chemotherapeutic agents have been associated with CIPN [2, 3] and existing clinical efforts aimed at preventing or treating CIPN remain disappointing due to their low efficacy [1].

The pathophysiology underlying CIPN is incompletely understood. Although chemotherapeutic agents preferentially act on rapidly proliferating tumor cells, post-mitotic cells such as peripheral sensory neurons of the dorsal root ganglion (DRG) are also susceptible to cell death which may be explained by the lack of a blood-brain barrier and the presence of an abundant fenestrated capillary network [2, 3]. Therefore, it is acknowledged that the absorption and accumulation of the drugs at the DRG contributes to CIPN [2, 4].

On top of the diverse array of clinical manifestations in CIPN patients [3] and in animal models [5], the multitude of intracellular processes targeted by cytotoxic chemotherapy further complicates a clear delineation of CIPN pathophysiology. Amidst the distinct mechanisms of action by different cytotoxic drugs and varying sensitivities of sensory neurons, there are still similarities, which suggest the existence of a common conserved effector mechanism [2, 3, 6, 7].

The protein c-Jun is a transcription factor of the activator protein-1 (AP-1) family that facilitates cellular stress response. Downstream effects of c-Jun activation include neuronal cell death, inflammation and regeneration [8–12]. Increased expression and activation of c-Jun have been observed in neurodegenerative disorders [13–15], nerve injury [16], as well as in neuropathies [17, 18]. The activation of c-Jun occurs when its N-terminal is phosphorylated by the c-Jun amino-terminal kinase (JNK) cascade and when its C-terminal is dephosphorylated by the extracellular signal regulated kinase (ERK) cascade [19].

For an in-depth investigation of c-Jun involvement in CIPN, we utilized induced pluripotent stem cell-derived sensory neurons (iPSC-DSN) as this model allows for large-scale in vitro production of sensory neurons of human origin to facilitate high-throughput experiments [20–23]. In this study, we treated iPSC-DSN with four different neurotoxic chemotherapeutic drugs, namely the taxane paclitaxel (PTX), the vinca alkaloid vincristine (VCR), the proteasome inhibitor bortezomib (BTZ) and the platinum compound cisplatin (CDDP) to elicit chemotherapy-induced neurotoxicity. To elucidate the role of c-Jun and investigate its implications in CIPN, c-Jun was inhibited by the application of a small molecule JNK inhibitor, SP600125 [24]. We evaluated multiple aspects of CIPN pathology in iPSC-DSN (viability, axon integrity, electrophysiology, transcriptome) to test our hypothesis that pharmacological inhibition of c-Jun phosphorylation mitigates neurotoxicity.

## Results

Human iPSC-DSN were characterized morphologically via immunofluorescence (IF) and functionally via live cell calcium imaging. The cells expressed typical markers of sensory neurons, such as peripherin, transient receptor potential vanilloid (TRPV) 1, TRPV4 and Na_v_1.7 channels, and generated calcium transients in response to chemical stimulation with capsaicin, icilin and ATP (Supplementary Fig. S1).

### Cytotoxic drugs upregulate phosphorylated c-Jun expression in iPSC-DSN

**Fig. 1:**
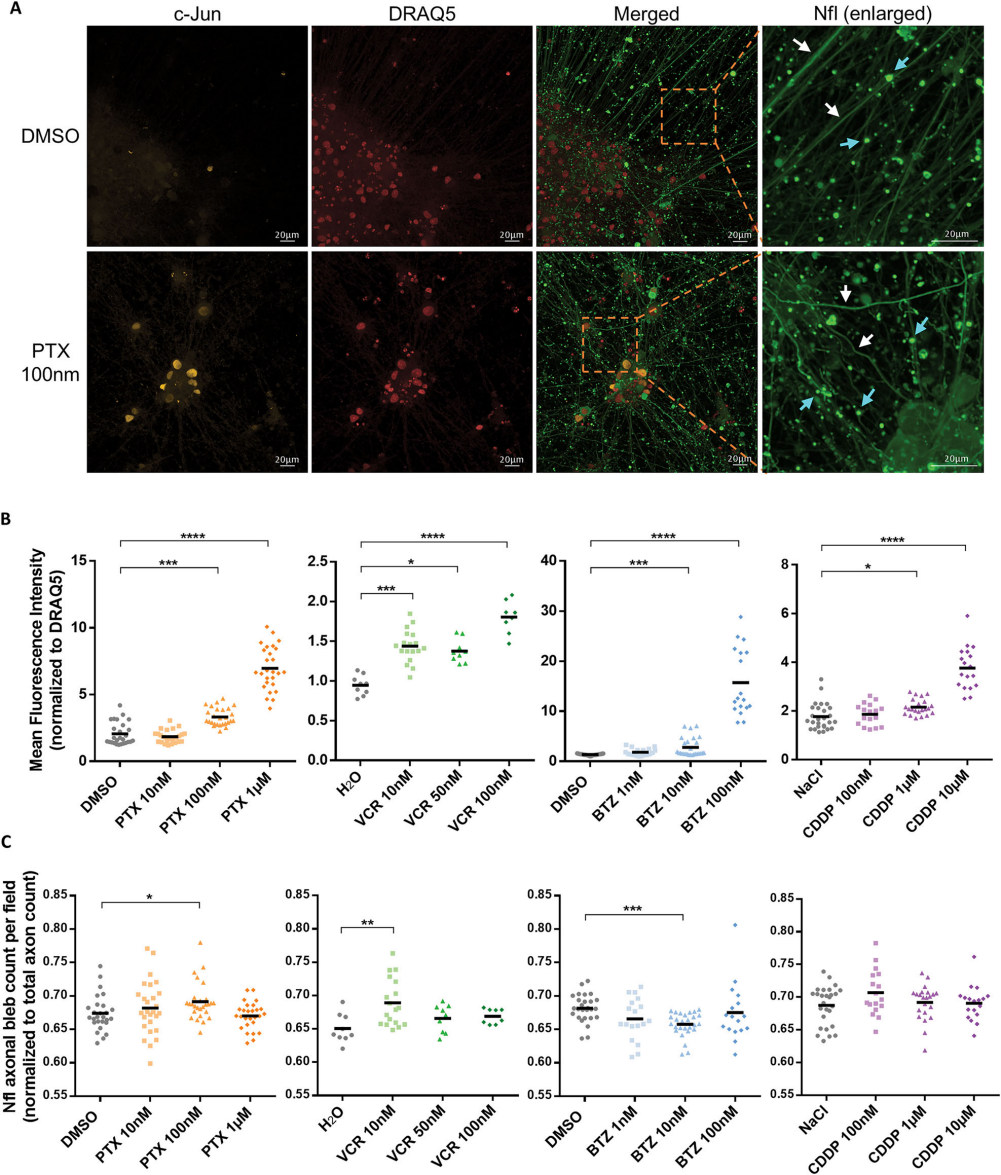
c-Jun expression and axonal blebs are increased when iPSC-DSN are incubated with cytotoxic drugs. **A** Representative immunofluorescent images of DMSO- or PTX-treated iPSC-DSN. After 72 h of treatment with 100 nM of PTX, the iPSC-DSN displayed increased c-Jun expression and compromised axon integrity in comparison to its vehicle counterpart (scale bar: 20 µm). Selected areas (of the neurofilament light chain layer), encased by dotted orange box, have been enlarged with axons marked by white arrows and blebs marked by cyan arrows. **B** Mean fluorescence intensity (MFI) of c-Jun normalized to MFI of a nuclear dye, DRAQ5, increases upon 72 h treatment with PTX, VCR, BTZ or CDDP (*n* = 27 in each group). **C** Neurofilament bleb count per field normalized to total axon count obtained via ImageJ increases upon 72 h treatment with 100 nM PTX or 10 nM VCR but decreases with 10 nM BTZ (*n* = 27 in each group). For **B** and **C**, data points from 3 wells per treatment condition and 9 fields per well were plotted. Statistical significance was assessed using the Kruskal–Wallis test, followed by Dunn’s multiple comparisons test. *p < 0.05, **p < 0.01, ***p < 0.001, ****p < 0.0001.

Mature iPSC-DSN (defined as iPSC-DSN cultivated for at least 40 days from the initiation of differentiation) treated with increasing concentrations of chemotherapy drugs (PTX, VCR, BTZ or CDDP) were imaged with confocal microscopy to observe for c-Jun expression and axonal damage (Fig. 1). At 72 h post-treatment, distinct morphological changes to axon integrity and heightened expression of c-Jun become visible (Fig. 1A). Further quantification revealed that c-Jun was upregulated by all chemotherapy drug types to varying degrees in a dose-dependent manner (Fig. 1B). PTX increased c-Jun expression at higher concentrations of 100 nM or 1 µM, which fall within the clinically applied dosage range, but not at a subtherapeutic dosage of 10 nM. VCR increased c-Jun at 10 nM, 50 nM and 100 nM while BTZ achieved upregulation at 10 nM and 100 nM. Meanwhile, this effect was noted in CDDP at the clinically applied dosages of 1–10 µM.

With the previous discovery of neurofilament proteins as meaningful biomarkers of CIPN [21], the morphology of neurofilament light chain (Nfl) structures was studied to assess axonal integrity. From the same set of confocal images, Nfl-positive blebs from disintegrated axons were quantified as a measure of axon degeneration (Fig. 1C). Analysis of axonal bleb to axon ratio revealed that, after 72 h of drug exposure, axonal blebbing was notably increased upon treatment with 100 nM of PTX, 10 nM of VCR, or 100 nM of CDDP, but decreased upon treatment with 10 nM of BTZ.

**Fig. 2:**
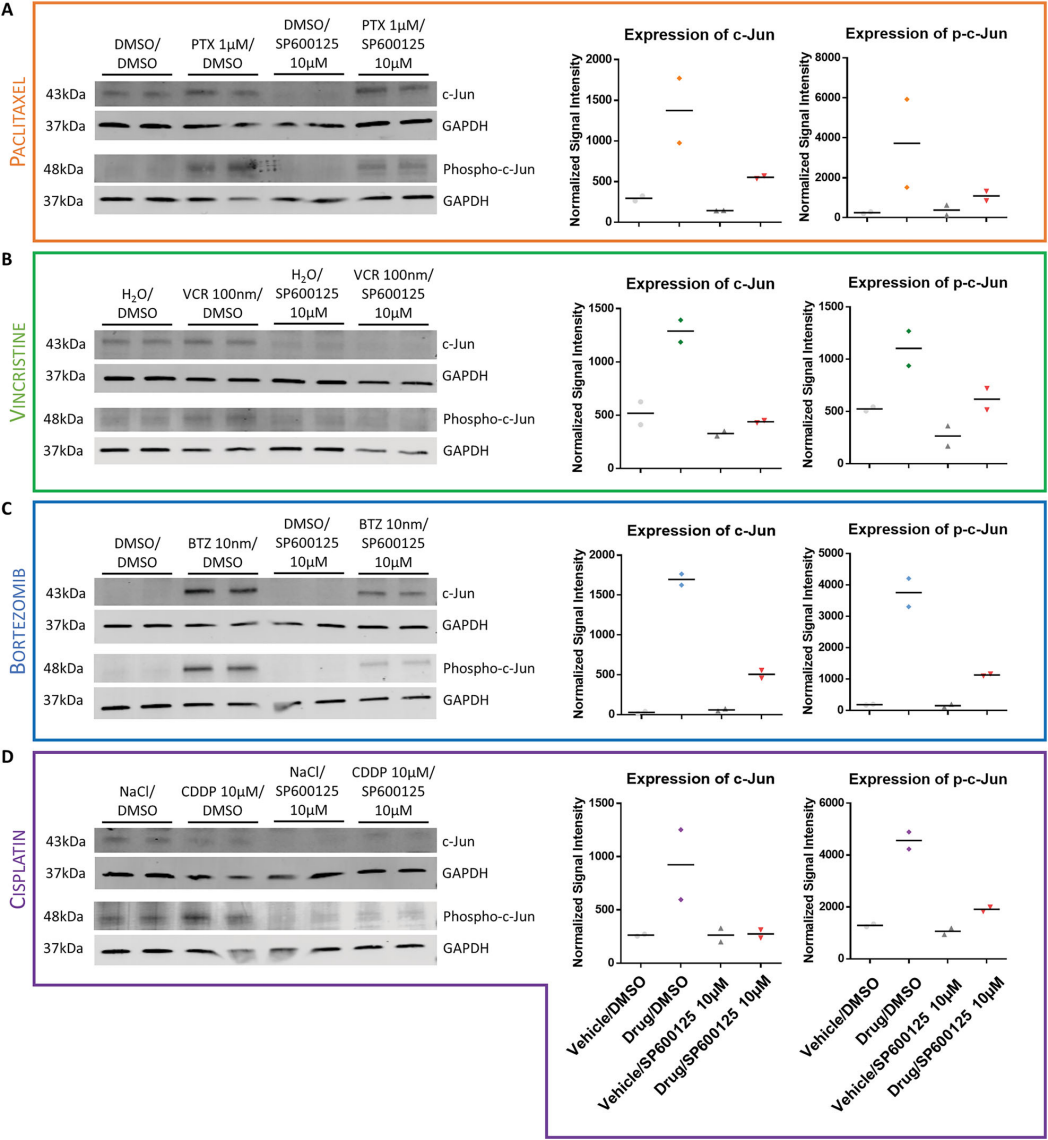
Upregulation of c-Jun expression and phosphorylation in iPSC-DSN upon treatment with cytotoxic drug is attenuated upon addition of SP600125. Western blots showing protein expression of c-Jun and phospho-c-Jun quantified by dot plots for iPSC-DSN treated with **A** PTX, **B** VCR, **C** BTZ or **D** CDDP, in comparison to vehicle control (DMSO), with and without SP600125 (*n* = 2 per treatment condition). Cell lysates of iPSC-DSN after 48-h incubation with PTX/BTZ/CDDP or 24-h incubation with VCR showed increased amounts of c-Jun and phospho-c-Jun, which was reduced in the presence of 10 µM SP600125. Signal intensities of the bands were normalized to housekeeping protein GAPDH and to their respective lanes.

Further investigation via western blot (WB) analysis was conducted to verify the expression levels as well as the activation state of c-Jun (Fig. 2). Clinically relevant dosages of PTX, VCR, BTZ and CDDP were used to treat iPSC-DSN and protein isolates were subsequently subjected to WB analysis. WB data confirmed that total c-Jun and total Ser73-phosphorylated c-Jun (p-c-Jun) levels were both increased upon treatment with the aforementioned cytotoxic drugs alone. Ratio of p-c-Jun/c-Jun was not calculated as the proteins were blotted on separate membranes due to limited availability of validated antibodies from different host species. To test whether this effect could be suppressed, cells were co-incubated with 10 µM of the small molecule inhibitor SP600125 (dose selected based on in vivo studies [10]). Results showed successful reduction of phosphorylated c-Jun levels across all four drug types (Fig. 2), thereby establishing SP600125-mediated c-Jun inhibition.

### c-Jun inhibition preserves viability and axon integrity of cytotoxin-treated iPSC-DSN

To monitor the effects of the inhibition of c-Jun phosphorylation on neurotoxicity in iPSC-DSN, a series of experiments was performed for each drug type in combination with SP600125. Cell viability was measured with a real-time non-lytic bioluminescent assay while confocal imaging was used to assess preservation of axonal integrity. In addition, microelectrode arrays (MEA) were conducted to examine the electrophysiological properties of iPSC-DSN. In each case, drug-treated samples were compared with their respective vehicle counterparts, with and without the presence of the small molecule inhibitor SP600125.

#### Paclitaxel

**Fig. 3:**
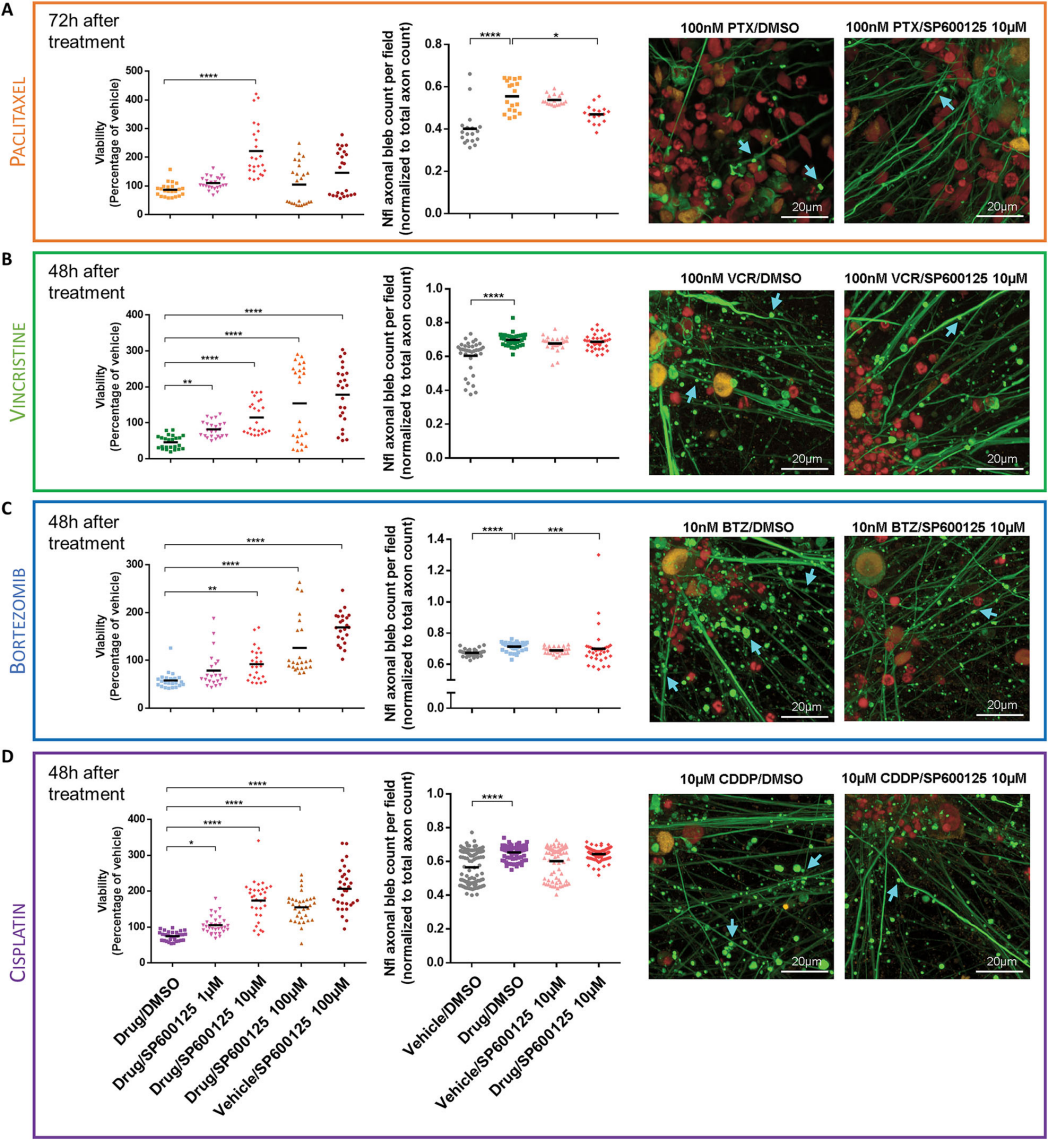
c-Jun inhibition improves iPSC-DSN cell viability and axon degeneration. iPSC-DSN were treated with **A** 100 nM PTX for 72 h, **B** 100 nM VCR for 48 h, **C** 10 nM BTZ for 48 h or **D** 10 µM CDDP for 48 h in conjunction with SP6000125 at increasing concentrations from 1 µM to 100 µM. Luminescence as a measure of cell viability was normalized to vehicle control (Vehicle/DMSO = 100%). Neurofilament axonal bleb count was obtained from immunofluorescent images using ImageJ and normalized to total axon count per field. Confocal images obtained from high content screening display neurofilament light chain in green, c-Jun in yellow and DRAQ5 in red (scale bar: 20 µm). Images have been enlarged and blebs annotated with cyan arrows. The original, uncropped images can be found in Supplementary Fig. S12. For the viability assay, 2 batches of differentiation (biological replicates) were seeded across ≥4 plates (technical replicates) with 6 wells per treatment condition (*n* = 24 for PTX, VCR and BTZ, *n* = 34 for CDDP). For axonal bleb quantification, ≥3 wells per treatment condition and ≥6 fields per well were imaged for each group (*n* = 18 for PTX, *n* = 36 for VCR and BTZ, *n* = 100 for CDDP). Statistical significance was determined using the Kruskal–Wallis test, followed by Dunn’s multiple comparisons test. *p < 0.05, **p < 0.01, ***p < 0.001, ****p < 0.0001.

iPSC-DSN that were exposed to 100 nM of PTX for 72 h in conjunction with 10 µM of SP600125 showed markedly improved viability (222% ± 93.8% of vehicle) in comparison to those exposed to 100 nM of PTX without SP600125 (86.8% ± 23.4% of vehicle, Kruskal–Wallis test, *p* < 0.0001; Fig. 3A). More subtle improvements were also observed when treated with 1 µM of SP600125 (110% ± 22.0% of vehicle) or with 100 µM of SP600125 (105% ± 72.1% of vehicle, Kruskal–Wallis test, *p* < 0.0001; Fig. 3A). The bleb to axon ratio increased upon treatment with 100 nM PTX (vehicle/DMSO: 0.402 ± 0.0893, 100 nM PTX/DMSO: 0.555 ± 0.0734, Kruskal–Wallis test, *p* < 0.0001; Fig. 3A) while addition of 10 µM SP600125 partially reversed this effect (100 nM PTX/DMSO: 0.555 ± 0.0734, 100 nM PTX/10 µM SP600125: 0.470 ± 0.0426, Kruskal–Wallis test, *p* < 0.05; Fig. 3A), suggesting a reduction of axonal degeneration. Replicating this assay on an alternate patient cell line showed similarly robust effects (Supplementary Fig. S2).

#### Vincristine

Addition of SP600125 improved cell viability at every dose (100 nM VCR/DMSO: 46.2% ± 18.6% of vehicle, 100 nM VCR/1 µM SP600125: 82.0% ± 22.2% of vehicle, 100 nM VCR/10 µM SP600125: 115% ± 45.5% of vehicle, 100 nM VCR/100 µM SP600125: 154% ± 105% of vehicle, Kruskal–Wallis test; Fig. 3B). Increased axonal blebbing was observed upon treatment with 100 nM VCR which was not alleviated by SP600125 (vehicle/DMSO: 0.604 ± 0.0962, 100 nM VCR/DMSO: 0.699 ± 0.0370, 100 nM VCR/10 µM SP600125: 0.687 ± 0.0421, Kruskal–Wallis test; Fig. 3B).

#### Bortezomib

In BTZ-treated iPSC-DSN, SP600125 improved cell viability at the 48-h timepoint in a dose-dependent manner (10 nM BTZ/DMSO: 58.2% ± 17.1% of vehicle, 10 nM BTZ/1 µM SP600125: 78.8% ± 36.8% of vehicle, 10 nM BTZ/10 µM SP600125: 92.2% ± 33.8% of vehicle, 10 nM BTZ/100 µM SP600125: 126% ± 61.1% of vehicle, Kruskal–Wallis test; Fig. 3C). Incubation with 10 nM of BTZ alone led to higher axonal bleb count (vehicle/DMSO: 0.674 ± 0.0215, 10 nM BTZ/DMSO: 0.714 ± 0.0285, Kruskal–Wallis test, *p* < 0.0001; Fig. 3C), which was ameliorated by co-incubation with 10 µM of SP600125 (10 nM BTZ/DMSO: 0.714 ± 0.0285, 10 nM BTZ/10 µM SP600125: 0.701 ± 0.124, Kruskal–Wallis test, *p* < 0.001; Fig. 3C), indicating preservation of axon integrity.

#### Cisplatin

Forty-eight hour exposure of iPSC-DSN to 10 µM of CDDP showed deteriorating cell viability that was reversed by the addition of 1 µM, 10 µM or 100 µM of SP600125 (10 µM CDDP/DMSO: 74.5% ± 12.9% of vehicle, 10 µM CDDP/1 µM SP600125: 106% ± 24.8% of vehicle, 10 µM CDDP/10 µM SP600125: 174% ± 54.4% of vehicle, 10 µM CDDP/100 µM SP600125: 155% ± 39.2% of vehicle, Kruskal–Wallis test; Fig. 3D). Meanwhile, 10 µM of CDDP increased axonal blebbing that was unaffected by SP600125 (vehicle/DMSO: 0.566 ± 0.0994, 10 µM CDDP/DMSO: 0.654 ± 0.0381, 10 µM CDDP/10 µM SP600125: 0.644 ± 0.0336, Kruskal–Wallis test; Fig. 3D).

Notably, iPSC-DSN cell lines tested in this (BIHi264-A, BIHi265-A) and in our previous studies (BIHi263-A, BIHi264-A, BIHi004-B, BIHi005-A) were all susceptible to neurotoxicity in vitro, regardless of whether the donor was a healthy control (BIHi004-B, BIHi005-A), or a chemotherapy patient who had developed CIPN (BIHi264-A, BIHi265-A) or not (BIHi263-A) [21, 25, 26].

Overall, cell viability of iPSC-DSN exposed to any of the studied cytotoxic drugs improved upon addition of SP600125 and axon degeneration induced by PTX or BTZ was reversed in the presence of SP600125. Importantly, SP600125 treatment on MCF7 breast cancer cells did not reduce antineoplastic efficacy of the drugs but suppressed proliferation from concentrations of 10 µM with a time- and dose-dependent decline of MCF7 viability (Supplementary Fig. S3). Cell viability of iPSC-DSN at all remaining timepoints (24 h/48 h/72 h) has been compiled in Supplementary Fig. S4.

### c-Jun inhibition ameliorates effects of chemotherapy on electrophysiology in iPSC-DSN

**Fig. 4:**
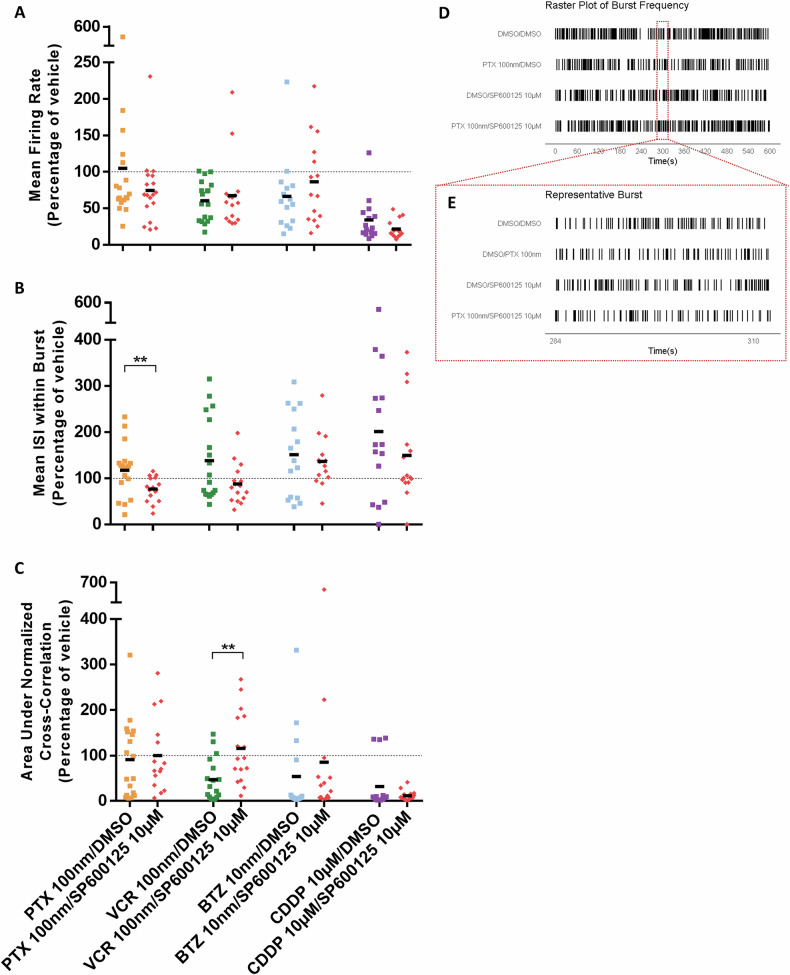
Electrophysiological response of iPSC-DSN from incubation with cytotoxic drugs in the presence of SP600125. Using MEA, a 10-min recording of the electrical activity of iPSC-DSN was obtained upon 24-h incubation with 100 nM PTX/100 nM VCR/10 nM BTZ/10 µM CDDP, in the presence and absence of 10 µM SP600125. Dot plots illustrate parameters of **A** Mean Firing Rate (MFR), **B** Mean Interspike Interval (ISI) within a burst and **C** Area Under Normalized Cross-correlation as a measure of synchronicity. Dotted line represents vehicle/DMSO = 100%. Raster plots depict the **D** burst frequency of iPSC-DSN over 10 min and **E** a representative example of spikes within a burst for each condition of PTX with SP600125. Data were obtained from triplicates of 6 wells per treatment condition for combined total of *n* = 18 per group. Statistical significance was evaluated with the Mann–Whitney *U* test (two-tailed). **p < 0.01.

Microelectrode array (MEA) recordings at 24-h post-treatment showed general decrease in Mean Firing Rate (MFR) of the iPSC-DSN when treated with most cytotoxic drugs (100 nM PTX: 105% ± 105% of vehicle, 100 nM VCR: 60.4% ± 27.3% of vehicle, 10 nM BTZ: 66.2% ± 50.3% of vehicle, 10 µM CDDP: 34.1% ± 29.6% of vehicle, Mann–Whitney *U* test, Fig. 4A). As a measure of action potentials per second [27], the MFR indicates the excitability of iPSC-DSN and these results demonstrate that iPSC-DSN treated with VCR, BTZ or CDDP without SP600125 had lower electrical activity in comparison to vehicle, whereas those treated with 100 nM PTX alone showed no significant change. Varying effects were observed when SP600125 was supplied. For PTX and for CDDP, the co-incubation with 10 µM SP600125 further reduces the MFR whereas for VCR and for BTZ, SP600125 increases the MFR (100 nM PTX/10 µM SP600125: 74.5% ± 47.2% of vehicle, 100 nM VCR/10 µM SP600125: 67.2% ± 48.1% of vehicle, 10 nM BTZ/10 µM SP600125: 86.3% ± 59.0% of vehicle, 10 µM CDDP/10 µM SP600125: 21.7% ± 12.2% of vehicle, Mann–Whitney *U* test, Fig. 4A).

Concurrently, changes in burst activity were monitored from the Mean Inter-spike Interval (ISI) within Burst, a metric which measures the time between spikes in a burst and reflects changes in burst activity caused by chemical alterations [27, 28]. All four chemotherapy drugs increased the mean ISI within a burst of the iPSC-DSN (100 nM PTX: 118% ± 56.1% of vehicle, 100 nM VCR: 138% ± 88.4% of vehicle, 10 nM BTZ: 151% ± 91.2% of vehicle, 10 µM CDDP: 201% ± 154% of vehicle, Mann–Whitney *U* test, Fig. 4B), indicating that the drugs disrupted burst activity by reducing the frequency of spikes (Fig. 4E). In the presence of 10 µM SP600125, a general trend could be observed where the Mean ISI within a burst was normalized (100 nM PTX/10 µM SP600125: 76.6% ± 26.3% of vehicle, 100 nM VCR/10 µM SP600125: 87.6% ± 42.4% of vehicle, 10 nM BTZ/10 µM SP600125: 137% ± 57.0% of vehicle, 10 µM CDDP/10 µM SP600125: 149% ± 105% of vehicle, Mann–Whitney *U* test, Fig. 4B).

The Area under Normalized Cross-correlation is a parameter that represents network connectivity and synchronicity [29, 30]. This parameter was reduced in the iPSC-DSN when exposed to any of the chemotherapy agents (100 nM PTX: 91.1% ± 85.1% of vehicle, 100 nM VCR: 47.1% ± 46.3% of vehicle, 10 nM BTZ: 53.8% ± 93.7% of vehicle, 10 µM CDDP: 32.1% ± 54.1% of vehicle, Mann–Whitney *U* test, Fig. 4C), suggesting that iPSC-DSN exposed to cytotoxic drug alone exhibited less synchrony. For all drugs except CDDP, c-Jun inhibition via SP600125 was able to normalize this effect in iPSC-DSN (100 nM PTX/10 µM SP600125: 100% ± 81.9% of vehicle, 100 nM VCR/10 µM SP600125: 116% ± 78.6% of vehicle, 10 nM BTZ/10 µM SP600125: 85.2% ± 167% of vehicle, 10 µM CDDP/10 µM SP600125: 11.7% ± 10.9% of vehicle, Mann–Whitney *U* test, Fig. 4C).

In summary, variations in the electrical response of iPSC-DSN to co-incubation of a cytotoxic drug with SP600125 were observed in Mean ISI within Burst and in synchronicity across the four drug types. Further timepoints (48 h/72 h) are given in Supplementary Fig. S5.

### c-Jun inhibition normalizes neuronal injury markers of iPSC-DSN following cytotoxic drug treatment

**Fig. 5:**
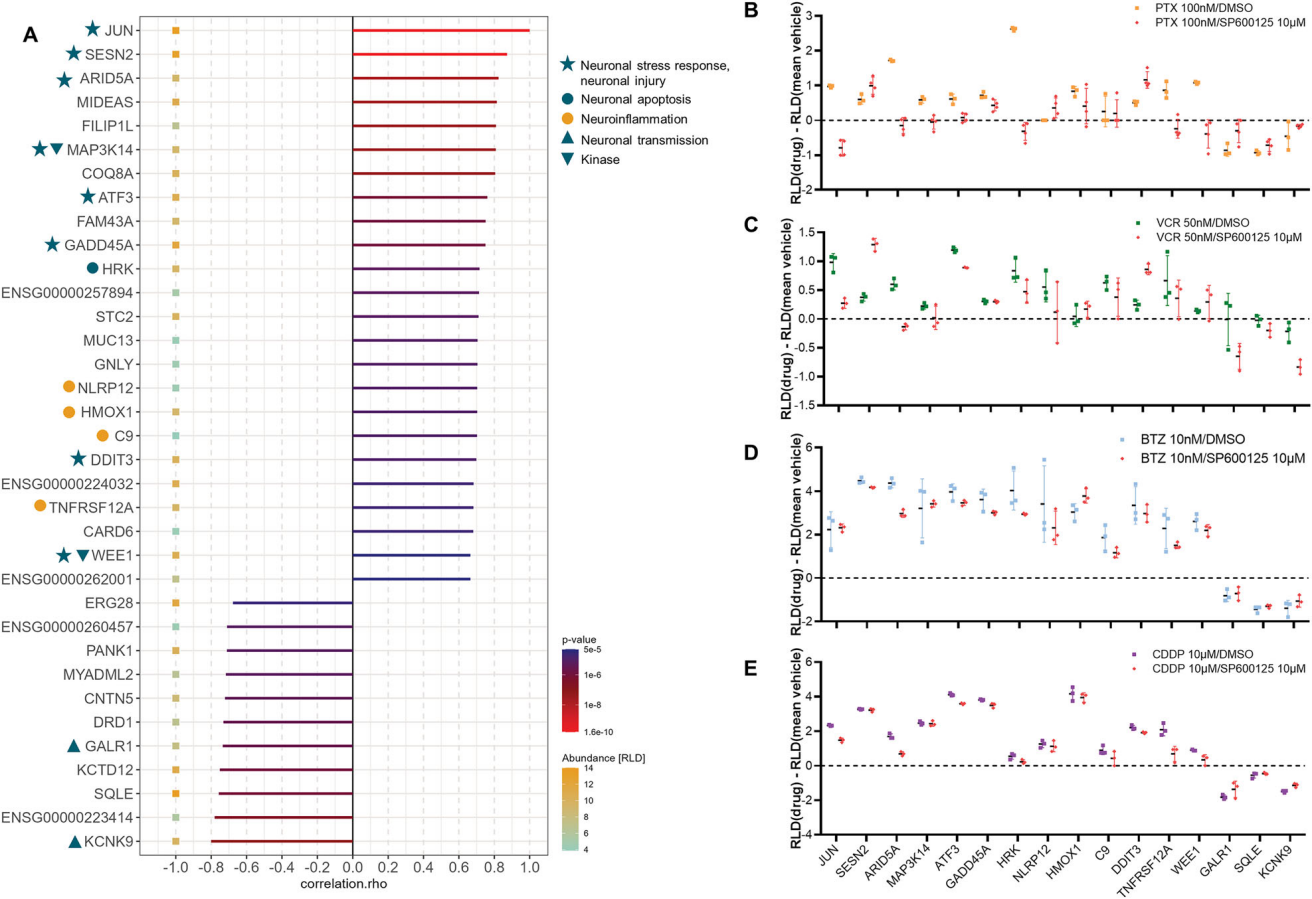
Transcriptomic analyses from iPSC-DSN treated with cytotoxic drug and SP600125. **A** Spearman correlation bar chart of JUN gene expression with the expression of selected genes (criteria, see below) in PTX/VCR/BTZ/CDDP- and vehicle-treated iPSC-DSN. mRNA expression levels of selected genes relative to vehicle from iPSC-DSN treated with **B** 100 nM PTX for 48 h, **C** 50 nM VCR for 16 h, **D** 10 nM BTZ for 16 h, or **E** 10 µM CDDP for 16 h, when SP600125 is present or absent (*n* = 3). Genes were sorted and selected based on overall Spearman correlation coefficient with reference to JUN, |R|, above 0.65, and based on whether they fulfilled the criterion of being differentially expressed with approximate absolute log2 fold change greater than 0.5 (RLD[drug] – RLD [mean vehicle] >0.5) in at least 3 out of 4 neurotoxic drugs. In **A**, 31 samples were included (drug and vehicle treated cells). In **B**–**E**, triplicates per condition were factored into the statistical analysis using the Mann–Whitney *U* test. Error bar represents the standard deviation of the mean.

To assess the downstream effects of c-Jun activation compared to its inhibition, RNA sequencing was performed on iPSC-DSN that were incubated with cytotoxic drugs or vehicle in the presence and absence of SP600125. Strong positive correlation of JUN to markers of neuronal stress response, injury and apoptosis (HRK, MAP3K14, GADD45A, WEE1), inflammation (ARID5A, NLRP12, C9, TNFRSF12A) and oxidative stress (HMOX1) were observed (≥0.65 Spearman correlation coefficient, Fig. 5A). There was also a strong negative correlation (≤−0.65) of JUN to markers of lipid metabolism (SQLE) and neuronal excitability (GALR1, KCNK9). Samples treated with any of the four cytotoxic drugs alone showed increased levels of JUN, ARID5A, MAP3K14, ATF3, GADD45A, HRK and TNFRSF12A, which were partially reversed when co-incubated with 10 µM SP600125 (Fig. 5B–E). For PTX-, BTZ- or CDDP-treated iPSC-DSN, the upregulation of WEE1 was attenuated by 10 µM SP600125 (Fig. 5B, D, E), while for VCR-, BTZ- or CDDP-treated iPSC-DSN, elevated levels of NRLP12 and C9 were reduced in the presence of SP600125 (Fig. 5C–E). In all samples, mRNA expression levels of GALR1, SQLE, KCNK9 were decreased (Fig. 5B–E) and only PTX-treated iPSC-DSN showed normalized effects with the addition of SP600125 (Fig. 5B).

JNK inhibition by 10 µM SP600125 (without chemotherapy) led to the expected downregulation of JUN as a direct phosphorylation target of JNK, and the differential expression of a few additional genes, such as MMP1, MMP10, SLC7A5, ASNSP1, MTHFD2, ATF4 and ATF5 (Supplementary Fig. S6).

## Discussion

Our study demonstrates that c-Jun is an important downstream mediator of neurotoxicity induced by several cytotoxic compounds that cause CIPN, i.e., PTX, VCR, BTZ and CDDP. Upon exposure to these neurotoxic agents, sensory neurons derived from iPSC show increased expression of phosphorylated c-Jun, followed by a decline in viability, axon integrity, burst activity and synchronicity. In contrast, pharmacological inhibition of c-Jun phosphorylation protected iPSC-DSN from the toxic effects of these compounds. Inhibition of c-Jun in each case yielded overall positive outcomes according to drug type and transcriptomic analyses strongly indicate that c-Jun is associated with overexpression of neuronal damage, neuroinflammation and apoptotic signatures that could be normalized by c-Jun inhibition.

The hypothesis that c-Jun is a common factor across different cytotoxic agents is further supported by the fact that upregulation of c-Jun and axon degeneration in iPSC-DSN occur together in all four chemotherapeutic agents tested. This result also confirms findings from animal models [17, 18] and in iPSC-DSN derived from different donors [21, 25]. The differential effects induced by each drug could be observed in various aspects. For example, BTZ displayed a time-dependent propensity for axonal degeneration, where increased blebbing was observed after 48 h of treatment but not after 72 h, showcasing that axonal blebbing is an early marker of axonal degeneration [31].

While phosphorylated c-Jun was attenuated by SP600125, expression of c-Jun itself was also downregulated despite SP600125 being a JNK-selective inhibitor. This can be attributed to an autoregulatory mechanism of c-Jun as the protein regulates the transcription of its own gene [32]. This finding is consistent with rodents, where decreased c-Jun mRNA levels were observed after loss of JNK signaling [33]. The existence of other cellular mechanisms that modulate c-Jun, such as ubiquitin-dependent c-Jun degradation mediated by COOH-terminal Src kinase [34], may further account for this JNK-independent c-Jun downregulation.

By using SP600125, we found that cell viability improved for iPSC-DSN incubated with any of the four cytotoxic drugs, reflecting the protective potential of c-Jun inhibition. Such viability results could consistently be replicated in an iPSC-DSN cell line of another individual, suggesting a robust finding (Supplementary Fig. S2). Interestingly, a very high dose of SP600125 (100 µM) did not improve cell viability in PTX-treated iPSC-DSN, contrary to the effect observed for the other three drug types. What sets PTX apart from the rest is likely a combination of its ability to stabilize microtubules without disrupting intracellular structures and the ability of SP600125 to promote the formation of polymerized tubulin [35], alluding to a potential synergistic cytotoxic effect of high-dose SP600125 with PTX.

Furthermore, the antineoplastic efficacy of chemotherapy in MCF7 breast cancer cells was maintained when co-incubated with cytotoxic drugs and SP600125 (Supplementary Fig. S3), suggesting that c-Jun inhibition with SP600125 preferentially protects postmitotic neuronal cells but not cancer cells. As pro-apoptotic mechanisms of JNK activation have been described in a pancreatic cancer cell line [36], preserved antineoplastic efficiency should be confirmed for different cancer types and treatments separately. The prospect of c-Jun targeted therapy in cancer treatment has gained increasing attention in recent literature [37–40] and is supported by our data that SP600125 decreases viability of MCF7 cells. Taken together, these observations build confidence in c-Jun as a potential therapeutic target in CIPN patients.

Nonetheless, it should be acknowledged that SP600125 has implications beyond JNK inhibition and its resultant c-Jun inhibition since SP600125 alone also positively influences iPSC-DSN viability. This suggests that there exist other pathways working in parallel with the c-Jun-mediated pathways. For instance, SP600125 has been found to activate the p38 MAPK pathway, which in turn stimulates the cAMP response element binding protein that is known to promote cell survival [41, 42]. In our study, potential off-target effects of SP600125 were uncovered from the differential expression of genes related to extracellular remodeling (MMP1, MMP10), the integrated neuronal stress response (ATF4, ATF5), amino acid transport and metabolism (SLC7A5, ASNSP1, MTHFD2), which may have partially contributed to the observed endpoints.

Axon degeneration was observed in all cytotoxin-treated iPSC-DSN. This is in accordance with previous literature where axonal transport impairments have been reported in drugs that do not directly affect microtubules, such as BTZ and CDDP [43–46]. Although SP600125 has been reported to promote tubulin polymerization in leukemia cells [35], our approach with SP600125 could not save axonal degeneration induced by every drug type; axon structures were only preserved in PTX- or BTZ-treated iPSC-DSN. This is interesting to note because it suggests that c-Jun is only partially associated with axon degeneration and regeneration. It is likely that other mechanisms are responsible, and c-Jun inhibition merely halts part of the cellular processes triggered by neurotoxic injury. Due to the heterogeneity of cells within the iPSC-DSN ganglia (Supplementary Fig. S7), it cannot be excluded that only specific subsets of neuronal cells (nociceptor/mechanoreceptor/proprioceptor) are affected by c-Jun. The inhibition of c-Jun phosphorylation has also been reported to reduce axonal outgrowth in rodent DRG [47], implying that this approach stops or slows down axon degeneration but does not promote regeneration.

The effects of blocking c-Jun phosphorylation were further examined at the electrophysiological level. In iPSC-DSN, c-Jun upregulation after exposure to cytotoxic agents generally coincided with reduced spontaneous activity, but differential effects of each drug were observed. For VCR, BTZ and CDDP, the reduced MFR after 24 h suggests neurotoxicity in the iPSC-DSN, coinciding with axon degeneration as supported by decreased synchronicity in MEA and morphological changes seen in parallel cultures on imaging plates. For PTX, the preserved MFR despite observed axon degeneration corroborates the reported ability of the ganglia to maintain or enhance spontaneous activity and excitability following PTX exposure [48, 49].

For sensory neurons, bursts and synchronous firing are crucial features of sensory processes for transmitting information [50–52]. Thus, impaired bursting and reduced synchrony would affect the iPSC-DSN’s ability to relay sensory signals. The burst activity of sensory neurons has also been documented to be controlled by either intrinsic cellular mechanisms or by inputs onto the dendrites [50]. Within the iPSC-DSN in vitro network, perhaps the impairment of their axons led to a loss of such inputs.

Overall, there could be many plausible reasons for electrophysiological abnormalities apart from axon degeneration, such as ion channel dysfunction, metabolic stress or synaptic impairment. Our findings of SP600125 counteracting the drugs’ cytotoxic effects and protecting the iPSC-DSN imply that their burst firing ability and synchronicity can be modulated by c-Jun/JNK-related pathways or even, to some extent, by the pleiotropic effects of SP600125.

Finally, we studied the effects of c-Jun inhibition on the transcriptome. Analysis at the RNA sequencing level revealed a strong correlation of JUN with neuronal stress, injury, inflammatory, metabolic and neuromodulation signatures among all drug types. In the iPSC-DSN exposed to any of the four cytotoxic drugs, we observed an upregulation of JUN and ATF3, which are genes of transcription factors involved in cellular stress response [18, 53], as well as an increase in SESN2 and HMOX1, which have been implicated in oxidative stress response pathways [54–56]. Differential expression of apoptosis or neurotransmission-related genes such as GADD45A [57, 58], HRK [10], C9 [59], DDIT3 [60] and GALR1 [61–63], was attenuated in the presence of SP600125, signaling a mechanistic role of c-Jun in mediating the processes contributing to CIPN. Inflammation may also be associated, as evidenced by upregulated ARID5A [64, 65] and TNFRSF12 [66] transcription. The interaction of ARID5A with IL-6 and Stat3 has even been reported to assist axonal regeneration [67, 68]. Consistently decreased KCNK9 levels suggest that regulation of two-pore potassium channels is involved in chemotherapy-induced neurotoxicity [69].

Arguably, one might contend that the focal point of CIPN pathophysiology could lie further upstream of c-Jun, for instance, in the JNK phosphorylation cascade. Reports of increased JNK phosphorylation in PTX-treated cancer cells [70] and in BTZ-treated rodent DRG [71–73] lean towards the notion that the ameliorative effects of SP600125 on iPSC-DSN treated with cytotoxic drugs are due to JNK. Even though transcriptomic data did not show elevated transcription levels of MAPK (the gene for JNK), the activation status of JNK depends on its phosphorylation state rather than its RNA expression levels. However, the activation state of c-Jun can also be altered by other proteins like ERK, p300 or GSK-3 [74] and, ultimately, the high correlation of JUN with neuronal injury markers in the transcriptomic data strongly suggests c-Jun to be the common point of convergence across all four chemotherapy agents.

In summary, our study identifies a central mechanistic role of c-Jun in the pathogenesis of CIPN. Through multiple approaches, we discovered that the inhibition of c-Jun phosphorylation confers protection to iPSC-DSN when treated with different neurotoxic drugs of varying mechanisms, thereby providing preclinical evidence that c-Jun mitigates chemotherapy-induced neurotoxicity and opening a promising avenue for targeted therapy. These findings also pave the way for future investigations into JNK-specific pathways or direct c-Jun modulations in the context of CIPN.

## Materials and methods

### Cell culture and differentiation

iPSCs were generated from two female donors (Berlin Institute of Health Core Unit Pluripotent Stem Cells and Organoids, BIHi264-A cell line, https://hpscreg.eu/cell-line/BIHi264-A and BIHi265-A cell line, https://hpscreg.eu/cell-line/BIHi265-A). The reprogramming and validation processes are detailed by Lewis et al. [26]. iPSCs were cultured in mTeSR™1 medium (Stem Cell Technologies) with full media change daily and passaged every 3–4 days upon reaching ~70% confluency with 0.5 mM EDTA (Gibco) diluted in 1x PBS without Ca^2+^ and Mg^2+^. Two to three days before differentiation, the iPSCs were detached with 1x TrypLE™ Select (Gibco) and single cell-seeded onto Geltrex (Gibco)-coated 6-well plates in mTeSR™1 medium supplemented with 10 μM Rock inhibitor (Stem Cell Technologies) at approximately 300,000 cells per well. The rock inhibitor was removed 24 h after seeding.

The differentiation protocol used was described by Huehnchen et al. [21]. In brief, differentiation of iPSCs into iPSC-DSN was conducted using small molecule inhibitors LDN193189 (Sigma), SB431542 (biogems), CHIR99021 (Sigma), DAPT (Sigma) and SU5402 (Sigma) across an 11-day period. Specifics on media composition and any further details on the differentiation, purification treatment and maintenance are provided in Supplementary Information. The exact timeline of differentiation has been outlined in Table 1 of Supplementary Information. Images confirming the purity of cultures can be found in Supplementary Fig. S8.

### Drug preparation

Upon maturation of iPSC-DSN (>40 days onwards), iPSC-DSNs were treated with varying concentrations of the chemotherapy drugs, paclitaxel (PTX, AdipoGen), vincristine (VCR, Cayman Chemical), bortezomib (BTZ, Cayman Chemical) or cisplatin (CDDP, Sigma) in addition to the JNK inhibitor SP600125 (Selleckchem). Stock solutions of PTX (6 mM), BTZ (6 mM) or SP600125 (48 mM) were prepared by reconstituting them in DMSO. VCR (6 mM) was dissolved in sterile distilled water while CDDP (6 mM) was dissolved in 0.9% NaCl after sonicating for 30 min–1 h at 37 °C. Final concentrations for the experiments were prepared by diluting stock solutions with N2B27 media with growth factors. For each experiment, the exact treatment concentrations and experimental timepoints can be found in the respective figure captions. A summary behind the rationale for selected timepoints can also be found in Supplementary Information in Table 2. The maximum concentration of DMSO in the well is 0.208% in co-incubated samples.

### Immunofluorescence (IF) and high content screening

iPSC-DSN seeded at 100,000 cells/cm^2^ onto black 24-well imaging plates (ibidi) were treated with drugs as mentioned above before fixation with 2% formaldehyde (Carl Roth) diluted in media for 15 min at room temperature. Samples were blocked with 10% normal goat serum, 1% bovine serum albumin and 0.1% Triton X in PBS with Ca^2+^ and Mg^2+^ for 1 h at room temperature before overnight incubation with primary antibodies in 1% BSA in PBS. Subsequently, samples were incubated with secondary antibodies in 1% BSA in PBS for 1 h at room temperature and then 0.01 mM DRAQ5 (Thermo Scientific) for 30 min at room temperature before imaging. Between each step, the wells were washed twice using PBS with Ca^2+^ and Mg^2+^. Detached wells were excluded from analysis. A list of the antibodies used can be found in Table 3 of the Supplementary Information. Confocal imaging was carried out using Revvity Opera Phenix.

### Image analysis

For each treatment condition, multiple fields (minimum 6) were imaged for 2–4 replicates which results in a minimum of 12 data points per condition. Images obtained from high content screening (HCS) were analyzed via ImageJ (Supplementary Fig. S9). Macros are provided in Supplementary Information. For the quantification of c-Jun expression, ImageJ-generated regions of interest measured the Mean Fluorescence Intensity of c-Jun and of DRAQ5. For axonal damage quantification, particle analysis on ImageJ counted Nfl spots in each image to obtain bleb counts, and the total Nfl area was defined by the total axon area. The total axon bleb count was normalized to the total axon count of each individual field to attain a metric of axon degeneration. Detached wells were excluded from analysis.

### Western blot and quantification

Full details for Western blot can be found in the Supplementary Information, including the antibody list in Table 3 and buffer formulas in Table 4. In brief, whole cell lysates were acquired from drug-treated mature iPSC-DSNs that were cultured on 6-well plates at 700,000–1,000,000 cells per well. PTX/BTZ/CDDP-treated samples were harvested after 48 h of treatment, while VCR-treated samples were harvested after 24 h. For fluorescent Western blot, proteins were loaded at 10 μg per well and separated on 4–15% polyacrylamide gels (Bio-Rad), then transferred onto polyvinylidene fluoride (PVDF) membranes (Millipore). These membranes were blocked with blocking buffer before incubation with primary antibodies, which were subsequently visualized with fluorescent-conjugated secondary antibodies. Scanned images were obtained with Li-Cor Odyssey® CLx Image and uncropped images can be found in Supplementary Fig. S10. Quantitative analysis was performed with Li-Cor Image Studio™ Lite. Fluorescent values of each band were normalized to their respective lane and to the housekeeping protein GAPDH.

### Live cell viability assay

iPSC-DSN were cultured at 48,000 cells/well in white (Greiner) or transparent (Falcon) flat, clear-bottomed 96-well plates and, upon maturation, treated with cytotoxic drugs in combination with 1 μM, 10 μM or 100 μM of SP600125. Reagents from RealTime-Glo™ MT Cell Viability Assay (Promega) were added according to the manufacturer’s protocol. Live luminescence readings were taken every 12 h up to 72 h with a microplate reader, Tristar LB941 (Berthold). A measure of cell viability, expressed as a percentage of vehicle, was obtained after normalizing raw luminescence values to individual wells and to their respective vehicle controls. Preselected wells were excluded before the experiments if morphological quality criteria were not met. The same assay was applied to MCF7 breast cancer cells and information on their media can be found in the Supplementary Information.

### Microelectrode array (MEA)

iPSC-DSN at 30,000 cells/well were seeded onto the electrodes of 48-well MEA plates (16 electrodes/well, Axion Biosystems, Supplementary Fig. S11). Specifics on MEA plate coating can be found in Supplementary Information. After 60 days of maturation, a baseline of electrophysiological activity was recorded in Maestro Pro (Axion Biosystems) before the cells were treated with the aforementioned drugs, after which measurements occurred every 6 h up to 72 h. Raw values obtained were normalized to the individual well baseline and to their respective vehicle controls.

### RNA extraction and sequencing

Mature iPSC-DSN from 6-well plates (700,000–1,000,000 cells/well) were treated with PTX for 48 h or VCR/BTZ/CDDP for 16 h, together with 10 µM of SP600125, prior to RNA extraction using Aurum™ Total RNA Mini Kit (Bio-Rad) according to the manufacturer’s instructions. RNA samples were sequenced by Brooks Life Sciences Genewiz® with PolyA selection for RNA removal, 2 × 150 bp sequencing configuration and 20–30 million reads per sample. RNA Seq reads were mapped to the human genome (GRCh38.p7) with STAR version 2.7.3a [75] using the default parameters. Reads were assigned to genes with featureCounts version 2.0.0 [76] with the following parameters: -t exon -g gene_id -s 0 -p, gene annotation - Gencode GRCh38/v25. The differential expression analysis was carried out with DESeq2 version 1.32.0 [77] using the default parameters. Gene set enrichment analysis was carried out with R/tmod package version 0.50.07 using MSigDB gene sets [78].

### Statistical analyses

Visualization and statistical analyses for IF HCS images, Western blot quantification, live cell viability assays, MEA and RNA sequencing were done with Prism 6 (GraphPad Software). As the Shapiro–Wilk normality test revealed that the data do not follow a normal distribution, nonparametric tests were chosen. For comparison between two groups, the Mann–Whitney *U* test was used, while for comparison across groups of more than three, the Kruskal–Wallis test corrected with Dunn’s multiple comparison test was used. Raster plots and Spearman correlations were generated using R.

All experiments were performed on iPSC-DSN from the BIHi264-A cell line unless otherwise specified. Biological replicates refer to samples from separate batches of differentiation, while technical replicates refer to repeat experiments, including samples from different cell culture wells or plates. In the IF and microscopy experiments for c-Jun expression and axonal bleb quantification, at least three wells (technical replicates) for each treatment condition were used. In the WB, multiple membranes were blotted for each drug type (technical replicates), and the average of two bands (technical replicates) was calculated. In the viability assay, two batches of differentiation (biological replicates) were each seeded onto two 96-well plates (technical replicates) for a total of four experimental plates per drug type. In the MEA experiment, two batches of differentiation (biological replicates) were distributed over six 48-well plates (technical replicates). In RNA sequencing, at least three samples (technical replicates) per treatment condition were analyzed, resulting in 48 samples. Exact sample sizes (*n*) for each experiment are stated in the respective figure legends.

## Supplementary information

Supplemental Information (Additional Methods and Tables)

Supplemental Figures with Captions

Dataset

Material List

Original Data (of Western blots)

## Data Availability

The full material list, including catalog numbers, all Excel spreadsheets, GraphPad Prism files and R scripts, is available as Supplementary Information. The RNA sequencing dataset generated from this study is available at NCBI’s Gene Expression Omnibus [79], under accession number GSE305998.

## Code availability

The R code for raster plots, Spearman correlation bar chart and the codes for ImageJ macros can be found in the Supplementary Information.

## References

[1] Prager K, Passig K, Micke O, Zomorodbakhsch B, Keinki C, Hubner J. Chemotherapy-induced polyneuropathy in cancer care—the patient perspective. Support Care Cancer. 2023;31:235. 36971861 10.1007/s00520-023-07688-5 PMC10042917

[2] Ibrahim EY, Ehrlich BE. Prevention of chemotherapy-induced peripheral neuropathy: a review of recent findings. Crit Rev Oncol Hematol. 2020;145:102831. 31783290 10.1016/j.critrevonc.2019.102831 PMC6982645

[3] Windebank AJ, Grisold W. Chemotherapy-induced neuropathy. J Peripher Nerv Syst. 2008;13:27–46. 18346229 10.1111/j.1529-8027.2008.00156.x

[4] Kim JH, Dougherty PM, Abdi S. Basic science and clinical management of painful and non-painful chemotherapy-related neuropathy. Gynecol Oncol. 2015;136:453–9. 25584767 10.1016/j.ygyno.2015.01.524 PMC5439219

[5] Boehmerle W, Huehnchen P, Peruzzaro S, Balkaya M, Endres M. Electrophysiological, behavioral and histological characterization of paclitaxel, cisplatin, vincristine and bortezomib-induced neuropathy in C57Bl/6 mice. Sci Rep. 2014;4:6370. 25231679 10.1038/srep06370 PMC5377307

[6] Yamamoto S, Egashira N. Pathological mechanisms of bortezomib-induced peripheral neuropathy. Int J Mol Sci. 2021;22:888. 33477371 10.3390/ijms22020888 PMC7830235

[7] Huehnchen P, Muenzfeld H, Boehmerle W, Endres M. Blockade of IL-6 signaling prevents paclitaxel-induced neuropathy in C57Bl/6 mice. Cell Death Dis. 2020;11:45. 31969555 10.1038/s41419-020-2239-0 PMC6976596

[8] Herdegen T, Skene P, Bähr M. The c-Jun transcription factor—bipotential mediator of neuronal death, survival and regeneration. Trends Neurosci. 1997;20:227–31. 9141200 10.1016/s0166-2236(96)01000-4

[9] Ham J, Eilers A, Whitfield J, Neame SJ, Shah B. c-Jun and the transcriptional control of neuronal apoptosis. Biochem Pharm. 2000;60:1015–21. 11007936 10.1016/s0006-2952(00)00372-5

[10] Ma C, Ying C, Yuan Z, Song B, Li D, Liu Y, et al. dp5/HRK is a c-Jun target gene and required for apoptosis induced by potassium deprivation in cerebellar granule neurons. J Biol Chem. 2007;282:30901–9. 17428807 10.1074/jbc.M608694200

[11] Feinberg K, Kolaj A, Wu C, Grinshtein N, Krieger JR, Moran MF, et al. A neuroprotective agent that inactivates prodegenerative TrkA and preserves mitochondria. J Cell Biol. 2017;216:3655–75. 28877995 10.1083/jcb.201705085 PMC5674898

[12] Hollville E, Romero SE, Deshmukh M. Apoptotic cell death regulation in neurons. FEBS J. 2019;286:3276–98. 31230407 10.1111/febs.14970 PMC6718311

[13] Okazawa H, Estus S. The JNK/c-Jun cascade and Alzheimer’s disease. Am J Alzheimers Dis Other Demen. 2002;17:79–88. 11954673 10.1177/153331750201700209 PMC10833950

[14] Akhter R, Sanphui P, Das H, Saha P, Biswas SC. The regulation of p53 up-regulated modulator of apoptosis by JNK/c-Jun pathway in β-amyloid-induced neuron death. J Neurochem. 2015;134:1091–103. 25891762 10.1111/jnc.13128

[15] Scopa C, Barnada SM, Cicardi ME, Singer M, Trotti D, Trizzino M. JUN upregulation drives aberrant transposable element mobilization, associated innate immune response, and impaired neurogenesis in Alzheimer’s disease. Nat Commun. 2023;14:8021. 38049398 10.1038/s41467-023-43728-8 PMC10696058

[16] Parkinson DB, Bhaskaran A, Arthur-Farraj P, Noon LA, Woodhoo A, Lloyd AC, et al. c-Jun is a negative regulator of myelination. J Cell Biol. 2008;181:625–37. 18490512 10.1083/jcb.200803013 PMC2386103

[17] Hutton EJ, Carty L, Laurá M, Houlden H, Lunn MPT, Brandner S, et al. c-Jun expression in human neuropathies: a pilot study. J Peripher Nerv Syst. 2011;16:295–303. 22176144 10.1111/j.1529-8027.2011.00360.x

[18] Liu C, Luan S, OuYang H, Huang Z, Wu S, Ma C, et al. Upregulation of CCL2 via ATF3/c-Jun interaction mediated the Bortezomib-induced peripheral neuropathy. Brain Behav Immun. 2016;53:96–104. 26554515 10.1016/j.bbi.2015.11.004

[19] Meng Q, Xia Y. c-Jun, at the crossroad of the signaling network. Protein Cell. 2011;2:889–98. 22180088 10.1007/s13238-011-1113-3 PMC4875184

[20] Schinke C, Fernandez Vallone V, Ivanov A, Peng Y, Körtvelyessy P, Nolte L, et al. Modeling chemotherapy induced neurotoxicity with human induced pluripotent stem cell (iPSC)-derived sensory neurons. Neurobiol Dis. 2021;155:105391. 33984509 10.1016/j.nbd.2021.105391

[21] Huehnchen P, Schinke C, Bangemann N, Dordevic AD, Kern J, Maierhof SK, et al. Neurofilament proteins as a potential biomarker in chemotherapy-induced polyneuropathy. JCI Insight. 2022;7:e154395. 35133982 10.1172/jci.insight.154395 PMC8986065

[22] Mortensen C, Thomsen MT, Chua KC, Hammer HS, Nielsen F, Potz O, et al. Modeling mechanisms of chemotherapy-induced peripheral neuropathy and chemotherapy transport using induced pluripotent stem cell-derived sensory neurons. Neuropharmacology. 2024;258:110062. 38972371 10.1016/j.neuropharm.2024.110062

[23] Odawara A, Shibata M, Ishibashi Y, Nagafuku N, Matsuda N, Suzuki I. In vitro pain assay using human iPSC-derived sensory neurons and microelectrode array. Toxicol Sci. 2022;188:131–41. 35478041 10.1093/toxsci/kfac045

[24] Bennett BL, Sasaki DT, Murray BW, Leary EC, Sakata ST, Xu W, et al. SP600125, an anthrapyrazolone inhibitor of Jun N-terminal kinase. Proc Natl Acad Sci USA. 2001;98:13681. 11717429 10.1073/pnas.251194298 PMC61101

[25] Schinke C, Fernandez Vallone V, Ivanov A, Peng Y, Körtvelyessy P, Nolte L, et al. Dataset for: modeling chemotherapy induced neurotoxicity with human induced pluripotent stem cell (iPSC)-derived sensory neurons. Data Brief. 2021;38:107320. 34485650 10.1016/j.dib.2021.107320 PMC8408513

[26] Lewis K, Vallone VF, Dordevic A, Kern J, Stachelscheid H, Endres M, et al. Generation of ten human induced pluripotent stem cell lines (hiPSCs) from patients with and without Chemotherapy-Induced Peripheral Neuropathy (CIPN) and Post Chemotherapy Cognitive Impairment (PCCI). Stem Cell Res. 2025;84:103674. 40015137 10.1016/j.scr.2025.103674

[27] Lv S, He E, Luo J, Liu Y, Liang W, Xu S, et al. Using human-induced pluripotent stem cell derived neurons on microelectrode arrays to model neurological disease: a review. Adv Sci. 2023;10:e2301828. 10.1002/advs.202301828 PMC10667858 37863819

[28] Fox DM, Rotstein HG, Nadim, F. Bursting in neurons and small networks. In: Jaeger D, Jung R. editors. Encyclopedia ofcomputational neuroscience. Springer, New York, NY. 2014. pp. 1–17. 10.1007/978-1-4614-7320-6_454-1.

[29] Suresh J, Radojicic M, Pesce LL, Bhansali A, Wang J, Tryba AK, et al. Network burst activity in hippocampal neuronal cultures: the role of synaptic and intrinsic currents. J Neurophysiol. 2016;115:3073–89. 26984425 10.1152/jn.00995.2015 PMC4946605

[30] Tukker AM, Wijnolts FMJ, de Groot A, Westerink RHS. Human iPSC-derived neuronal models for in vitro neurotoxicity assessment. Neurotoxicology. 2018;67:215–25. 29909083 10.1016/j.neuro.2018.06.007

[31] Faris H, Almasieh M, Levin LA. Axonal degeneration induces distinct patterns of phosphatidylserine and phosphatidylethanolamine externalization. Cell Death Discov. 2021;7:247. 34535640 10.1038/s41420-021-00641-7 PMC8448818

[32] Angel P, Hattori K, Smeal T, Karin M. The jun proto-oncogene is positively autoregulated by its product, Jun/AP-1. Cell. 1988;55:875–85. 3142689 10.1016/0092-8674(88)90143-2

[33] Sancho R, Nateri AS, de Vinuesa AG, Aguilera C, Nye E, Spencer-Dene B, et al. JNK signalling modulates intestinal homeostasis and tumourigenesis in mice. EMBO J. 2009;28:1843–54. 19521338 10.1038/emboj.2009.153 PMC2711188

[34] Zhu F, Choi BY, Ma W-Y, Zhao Z, Zhang Y, Cho YY, et al. COOH-terminal Src kinase–mediated c-Jun phosphorylation promotes c-Jun degradation and inhibits cell transformation. Cancer Res. 2006;66:5729–36. 16740711 10.1158/0008-5472.CAN-05-4466 PMC2239244

[35] Moon D-O, Kim M-O, Kang C-H, Lee J-D, Choi YH, Kim G-Y. JNK inhibitor SP600125 promotes the formation of polymerized tubulin, leading to G2/M phase arrest, endoreduplication, and delayed apoptosis. Exp Mol Med. 2009;41:665. 19478553 10.3858/emm.2009.41.9.073 PMC2753660

[36] Nawrocki ST, Carew JS, Pino MS, Highshaw RA, Dunner K, Huang P, et al. Bortezomib sensitizes pancreatic cancer cells to endoplasmic reticulum stress-mediated apoptosis. Cancer Res. 2005;65:11658–66. 16357177 10.1158/0008-5472.CAN-05-2370

[37] Brennan A, Leech JT, Kad NM, Mason JM. Selective antagonism of cJun for cancer therapy. J Exp Clin Cancer Res. 2020;39:184. 32917236 10.1186/s13046-020-01686-9 PMC7488417

[38] Han Y, Katayama S, Futakuchi M, Nakamichi K, Wakabayashi Y, Sakamoto M, et al. Targeting c-Jun is a potential therapy for luminal breast cancer bone metastasis. Mol Cancer Res. 2023;21:908–21. 37310848 10.1158/1541-7786.MCR-22-0695

[39] Yu P, Wei H, Li K, Zhu S, Li J, Chen C, et al. The traditional Chinese medicine monomer Ailanthone improves the therapeutic efficacy of anti-PD-L1 in melanoma cells by targeting c-Jun. J Exp Clin Cancer Res. 2022;41:346. 36522774 10.1186/s13046-022-02559-z PMC9753288

[40] Miao R, Dai C-C, Mei L, Xu J, Sun S-W, Xing Y-L, et al. KIAA1429 regulates cell proliferation by targeting c-Jun messenger RNA directly in gastric cancer. J Cell Physiol. 2020;235:7420–32. 32052427 10.1002/jcp.29645

[41] Vaishnav D, Jambal P, Reusch JEB, Pugazhenthi S. SP600125, an inhibitor of c-jun N-terminal kinase, activates CREB by a p38 MAPK-mediated pathway. Biochem Biophys Res Commun. 2003;307:855–60. 12878189 10.1016/s0006-291x(03)01287-7

[42] Finkbeiner S. CREB couples neurotrophin signals to survival messages. Neuron. 2000;25:11–14. 10707967 10.1016/s0896-6273(00)80866-1

[43] Klein I, Lehmann HC. Pathomechanisms of paclitaxel-induced peripheral neuropathy. Toxics. 2021;9:229. 34678925 10.3390/toxics9100229 PMC8540213

[44] LaPointe NE, Morfini G, Brady ST, Feinstein SC, Wilson L, Jordan MA. Effects of eribulin, vincristine, paclitaxel and ixabepilone on fast axonal transport and kinesin-1 driven microtubule gliding: implications for chemotherapy-induced peripheral neuropathy. Neurotoxicology. 2013;37:231–9. 23711742 10.1016/j.neuro.2013.05.008 PMC4169189

[45] Staff NP, Podratz JL, Grassner L, Bader M, Paz J, Knight AM, et al. Bortezomib alters microtubule polymerization and axonal transport in rat dorsal root ganglion neurons. Neurotoxicology. 2013;39:124–31. 24035926 10.1016/j.neuro.2013.09.001 PMC3844018

[46] Yan F, Liu JJ, Ip V, Jamieson SM, McKeage MJ. Role of platinum DNA damage-induced transcriptional inhibition in chemotherapy-induced neuronal atrophy and peripheral neurotoxicity. J Neurochem. 2015;135:1099–112. 26364854 10.1111/jnc.13355

[47] Lindwall C, Dahlin L, Lundborg G, Kanje M. Inhibition of c-Jun phosphorylation reduces axonal outgrowth of adult rat nodose ganglia and dorsal root ganglia sensory neurons. Mol Cell Neurosci. 2004;27:267–79. 15519242 10.1016/j.mcn.2004.07.001

[48] Zhang H, Dougherty PM. Enhanced excitability of primary sensory neurons and altered gene expression of neuronal ion channels in dorsal root ganglion in paclitaxel-induced peripheral neuropathy. Anesthesiology. 2014;120:1463–75. 24534904 10.1097/ALN.0000000000000176 PMC4031279

[49] Akin EJ, Alsaloum M, Higerd GP, Liu S, Zhao P, Dib-Hajj FB, et al. Paclitaxel increases axonal localization and vesicular trafficking of Nav1.7. Brain. 2021;144:1727–37. 33734317 10.1093/brain/awab113 PMC8320304

[50] Krahe R, Gabbiani F. Burst firing in sensory systems. Nat Rev Neurosci. 2004;5:13–23. 14661065 10.1038/nrn1296

[51] Chen L, Deng Y, Luo W, Wang Z, Zeng S. Detection of bursts in neuronal spike trains by the mean inter-spike interval method. Prog Nat Sci. 2009;19:229–35.

[52] Engel AK, Fries P, Singer W. Dynamic predictions: oscillations and synchrony in top–down processing. Nat Rev Neurosci. 2001;2:704–16. 11584308 10.1038/35094565

[53] Hai T, Wolfgang CD, Marsee DK, Allen AE, Sivaprasad U. ATF3 and stress responses. Gene Expr. 1999;7:321. 10440233 PMC6174666

[54] Kallenborn-Gerhardt W, Lu R, Syhr KM, Heidler J, von Melchner H, Geisslinger G, et al. Antioxidant activity of sestrin 2 controls neuropathic pain after peripheral nerve injury. Antioxid Redox Signal. 2013;19:2013–23. 23495831 10.1089/ars.2012.4958 PMC3869453

[55] Nitti M, Piras S, Brondolo L, Marinari UM, Pronzato MA, Furfaro AL. Heme oxygenase 1 in the nervous system: does it favor neuronal cell survival or induce neurodegeneration?. Int J Mol Sci. 2018;19:2260. 30071692 10.3390/ijms19082260 PMC6121636

[56] Chen K, Gunter K, Maines MD. Neurons overexpressing heme oxygenase-1 resist oxidative stress-mediated cell death. J Neurochem. 2000;75:304–13. 10854275 10.1046/j.1471-4159.2000.0750304.x

[57] Huang M, Wang J, Liu W, Zhou H. Advances in the role of the GADD45 family in neurodevelopmental, neurodegenerative, and neuropsychiatric disorders. Front Neurosci. 2024;18:1349409. 38332860 10.3389/fnins.2024.1349409 PMC10850240

[58] Wang XF, Zeng QG, Zeng Y, Man RY, Lu BX, Luo YF. Induction of GADD45α protects M17 neuroblastoma cells against MPP. IUBMB Life. 2014;66:786–92. 25469469 10.1002/iub.1327

[59] Schultz SJ, Aly H, Hasanen BM, Khashaba MT, Lear SC, Bendon RW, et al. Complement component 9 activation, consumption, and neuronal deposition in the post-hypoxic–ischemic central nervous system of human newborn infants. Neurosci Lett. 2005;378:1–6. 15763162 10.1016/j.neulet.2004.12.008

[60] Syc-Mazurek SB, Fernandes KA, Wilson MP, Shrager P, Libby RT. Together JUN and DDIT3 (CHOP) control retinal ganglion cell death after axonal injury. Mol Neurodegener. 2017;12:71. 28969695 10.1186/s13024-017-0214-8 PMC5625643

[61] Shi H, Fang Y, Huang L, Gao L, Lenahan C, Okada T, et al. Activation of galanin receptor 1 with M617 attenuates neuronal apoptosis via ERK/GSK-3β/TIP60 pathway after subarachnoid hemorrhage in rats. Neurotherapeutics. 2021;18:1905–21. 34086200 10.1007/s13311-021-01066-x PMC8609084

[62] Li S-Y, Huo M-L, Wu X-Y, Huang Y-Q, Wang L, Zhang X, et al. Involvement of galanin and galanin receptor 1 in nociceptive modulation in the central nucleus of amygdala in normal and neuropathic rats. Sci Rep. 2017;7:15317. 29127424 10.1038/s41598-017-13944-6 PMC5681679

[63] Stevenson L, Allen WL, Turkington R, Jithesh PV, Proutski I, Stewart G, et al. Identification of galanin and its receptor GalR1 as novel determinants of resistance to chemotherapy and potential biomarkers in colorectal cancer. Clin Cancer Res. 2012;18:5412–26. 22859720 10.1158/1078-0432.CCR-12-1780 PMC3463501

[64] Masuda K, Ripley B, Nishimura R, Mino T, Takeuchi O, Shioi G, et al. Arid5a controls IL-6 mRNA stability, which contributes to elevation of IL-6 level in vivo. Proc Natl Acad Sci USA. 2013;110:9409–14. 23676272 10.1073/pnas.1307419110 PMC3677444

[65] Masuda K, Ripley B, Nyati KK, Dubey PK, Zaman MM-U, Hanieh H, et al. Arid5a regulates naive CD4+ T cell fate through selective stabilization of Stat3 mRNA. J Exp Med. 2016;213:605–19. 27022145 10.1084/jem.20151289 PMC4821647

[66] Huang LN, Zou Y, Wu SG, Zhang HH, Mao QX, Li JB, et al. Fn14 participates in neuropathic pain through NF-kappaB pathway in primary sensory neurons. Mol Neurobiol. 2019;56:7085–96. 30976982 10.1007/s12035-019-1545-y PMC6728171

[67] Leibinger M, Zeitler C, Gobrecht P, Andreadaki A, Gisselmann G, Fischer D. Transneuronal delivery of hyper-interleukin-6 enables functional recovery after severe spinal cord injury in mice. Nat Commun. 2021;12:391. 33452250 10.1038/s41467-020-20112-4 PMC7810685

[68] Wareham LK, Echevarria FD, Sousa JL, Konlian DO, Dallas G, Formichella CR, et al. Interleukin-6 promotes microtubule stability in axons via Stat3 protein–protein interactions. iScience. 2021;24:103141. 34646984 10.1016/j.isci.2021.103141 PMC8496173

[69] Goldstein SAN, Bockenhauer D, O’Kelly I, Zilberberg N. Potassium leak channels and the KCNK family of two-p-domain subunits. Nat Rev Neurosci. 2001;2:175–84. 11256078 10.1038/35058574

[70] Meshkini A, Yazdanparast R. Involvement of oxidative stress in taxol-induced apoptosis in chronic myelogenous leukemia K562 cells. Exp Toxicol Pathol. 2012;64:357–65. 21074392 10.1016/j.etp.2010.09.010

[71] Zhang J, Su Y-M, Li D, Cui Y, Huang Z-Z, Wei J-Y, et al. TNF-α-mediated JNK activation in the dorsal root ganglion neurons contributes to Bortezomib-induced peripheral neuropathy. Brain Behav Immun. 2014;38:185–91. 24530998 10.1016/j.bbi.2014.01.020

[72] Liu D, Sun M, Xu D, Ma X, Gao D, Yu H. Inhibition of TRPA1 and IL-6 signal alleviates neuropathic pain following chemotherapeutic bortezomib. Physiol Res. 2019;68:845–55. 31424261 10.33549/physiolres.934015

[73] Li C, Deng T, Shang Z, Wang D, Xiao Y. Blocking TRPA1 and TNF-α signal improves bortezomib-induced neuropathic pain. Cell Physiol Biochem. 2018;51:2098–110. 30522101 10.1159/000495828

[74] Raivich G. c-Jun expression, activation and function in neural cell death, inflammation and repair. J Neurochem. 2008;107:898–906. 18793328 10.1111/j.1471-4159.2008.05684.x

[75] Dobin A, Davis CA, Schlesinger F, Drenkow J, Zaleski C, Jha S, et al. STAR: ultrafast universal RNA-seq aligner. Bioinformatics. 2013;29:15–21. 23104886 10.1093/bioinformatics/bts635 PMC3530905

[76] Liao Y, Smyth GK, Shi W. featureCounts: an efficient general purpose program for assigning sequence reads to genomic features. Bioinformatics. 2014;30:923–30. 24227677 10.1093/bioinformatics/btt656

[77] Love MI, Huber W, Anders S. Moderated estimation of fold change and dispersion for RNA-seq data with DESeq2. Genome Biol. 2014;15:550. 25516281 10.1186/s13059-014-0550-8 PMC4302049

[78] Liberzon A, Birger C, Thorvaldsdóttir H, Ghandi M, Mesirov JP, Tamayo P. The molecular signatures database hallmark gene set collection. Cell Syst. 2015;1:417–25. 26771021 10.1016/j.cels.2015.12.004 PMC4707969

[79] Edgar R, Domrachev M, Lash AE. Gene Expression Omnibus: NCBI gene expression and hybridization array data repository. Nucleic Acids Res. 2002;30:207–10. 11752295 10.1093/nar/30.1.207 PMC99122

