# Supplementary material for: c-Jun inhibition mitigates chemotherapy-induced neurotoxicity in iPSC-derived sensory neurons
Source: Cell Death Discov. 2025 Nov 13;11:529. doi: 10.1038/s41420-025-02847-5 (PMC12615653; doi:10.1038/s41420-025-02847-5)
Supplement: Supplementary file 1 — Supplemental Information (Additional Methods and Tables) [file 41420_2025_2847_MOESM1_ESM.docx]

**Additional Materials and Methods**

**Differentiation of iPSCs into iPSC-DSN**

Differentiation began on Day 0 when iPSCs are 50-60% confluent; mTeSR™1 maintenance media was replaced with knockout serum replacement (KSR) media which consisted of 20% CTS™ KnockOut™ SR XenoFree Medium (Gibco), 1x MEM Non-Essential Amino Acids (Gibco), 1x GlutaMAX™ Supplement (Gibco) and 0.01mM β-mercaptoethanol (Gibco). N2B27 media made from neurobasal media (Gibco) supplemented with 1x N2 (Gibco), 1x B27 without vitamin A (Gibco), 1x GlutaMAX™ Supplement (Gibco) and 0.01mM β-mercaptoethanol (Gibco) was gradually phased in from Day 4 to Day 11. Small molecule inhibitors 100nM LDN-193189 (Sigma) and 10µM SB-431542 (PeproTech) were supplemented from Day 0 to Day 5 while 3µM CHIR99021 (Sigma), 10µM DAPT (Sigma) and 10µM SU5402 (Sigma) were supplemented from Day 2 to Day 10. On Day 11, the differentiated cells are detached using 1x TrypLE™ Select (Gibco), filtered through a 40µM cell strainer (Falcon) and subsequently reseeded onto different Geltrex-coated cell culture vessels for downstream experiments. The timeline for the differentiation is summarized in *Table 1*. Three 6-well plates of iPSCs for this cell line produced approximately 222 million iPSC-DSNs at the end of differentiation. 10μM Rock inhibitor supplemented on Day 11 for reseeding was removed on Day 12. 1µg/ml mitomycin C (Sigma) treatment was conducted on Day 14 to eliminate non-neuronal dividing cells. This treatment was repeated once per batch as necessary for a maximum total of two treatments between Day 14 and Day 40. Half media change was conducted every 3-4 days for the iPSC-DSNs that were maintained in N2B27 media supplemented with β-nerve growth factor (β-NGF, PeproTech), brain derived neurotrophic factor (BDNF, PeproTech), neurotrophin 3 (NT3, PeproTech) and glial derived neurotrophic factor (GDNF, PeproTech) at 25ng/ml each as well as 100U/ml of Penicillin-Streptomycin (Gibco) or 1x Antibiotic-Antimycotic (Gibco).

**ImageJ Macros**

| For c-Jun quantification |
| --- |
| // Define the directories  inputDir = getDirectory("Choose Input Directory"); // Directory to open files from  outputDir = getDirectory("Choose Output Directory"); // Directory to save the data  // List all image files in the input directory  imageFiles = getFileList(inputDir);  // Loop through all image files  for (i = 0; i < imageFiles.length; i++) {  // Step 1: Open the image file  open(inputDir + imageFiles[i]);  originalTitle = getTitle(); // Store the original title for later use  // Remove everything after the first dot to clean up the file name  baseName = substring(originalTitle, 0, indexOf(originalTitle, "."));  // Step 2: Process the image  run("Duplicate...", "title=Processed");  selectWindow("Processed");  // Preprocessing: Enhance bright spots  run("Bandpass Filter...", "filter_large=40 filter_small=3 suppress=None tolerance=5 autoscale saturate");  run("Gaussian Blur...", "sigma=2");  // Thresholding: Isolate bright spots & Watershed to delineate clusters  setAutoThreshold("Triangle dark");  setThreshold(50629, 65535);  run("Convert to Mask");  run("Watershed");  // Particle Analysis: Focus on spots  run("Analyze Particles...", "size=20-Infinity circularity=0.50-1.00 add"); // Adjust size and circularity  // Step 3: Apply the ROIs to the original image  selectWindow(originalTitle); // Go back to the original image  roiManager("Show All");  roiManager("multi-measure measure_all"); // Measure all ROIs on the original image  // Step 4: Save the measurement data as an Excel file  saveAs("Results", outputDir + baseName + "_cJun_rawdata.xls");  // Close all images and reset ROI Manager  run("Close All");  run("Clear Results");  roiManager("Reset");  } |

| For DRAQ5 quantification |
| --- |
| // Define the directories  inputDir = getDirectory("Choose Input Directory"); // Directory to open files from  outputDir = getDirectory("Choose Output Directory"); // Directory to save the data  // List all image files in the input directory  imageFiles = getFileList(inputDir);  // Loop through all image files  for (i = 0; i < imageFiles.length; i++) {  // Step 1: Open the image file  open(inputDir + imageFiles[i]);  originalTitle = getTitle(); // Store the original title for later use  // Remove everything after the first dot to clean up the file name  baseName = substring(originalTitle, 0, indexOf(originalTitle, "."));  // Step 2: Process the image  run("Duplicate...", "title=Processed");  selectWindow("Processed");  // Preprocessing: Enhance bright spots  run("Bandpass Filter...", "filter_large=40 filter_small=3 suppress=None tolerance=5 autoscale saturate");  run("Gaussian Blur...", "sigma=2");  // Thresholding: Isolate bright spots & Watershed to delineate clusters  setAutoThreshold("Triangle dark");  run("Convert to Mask");  run("Watershed");  // Particle Analysis: Focus on spots  run("Analyze Particles...", "size=20-Infinity circularity=0.50-1.00 add"); // Adjust size and circularity  // Step 3: Apply the ROIs to the original image  selectWindow(originalTitle); // Go back to the original image  roiManager("Show All");  roiManager("multi-measure measure_all"); // Measure all ROIs on the original image  // Step 4: Save the measurement data as an Excel file  saveAs("Results", outputDir + baseName + "_DRAQ5_rawdata.xls");  // Close all images and reset ROI Manager  run("Close All");  run("Clear Results");  roiManager("Reset");  } |

| For Nfl-stained axon blebbing |
| --- |
| // Define the directories  inputDir = getDirectory("Choose Input Directory"); // Directory to open files from  outputDir = getDirectory("Choose Output Directory"); // Directory to save the data  // List all image files in the input directory  imageFiles = getFileList(inputDir);  // Loop through all image files  for (i = 0; i < imageFiles.length; i++) {  // Open the image file  open(inputDir + imageFiles[i]);  originalTitle = getTitle(); // Store the original title for later use  // Remove everything after the first dot to clean up the file name  baseName = substring(originalTitle, 0, indexOf(originalTitle, "."));  // Process the image  run("Duplicate...", "title=Processed");  selectWindow("Processed");  // Preprocessing: Enhance bright spots  run("8-bit");  // Thresholding: Isolate bright spots & Watershed to delineate clusters  setAutoThreshold("Li dark");  run("Convert to Mask");  // Particle Analysis  run("Analyze Particles...", "size=0-Infinity clear summarize"); // Total axons  run("Watershed");  run("Analyze Particles...", "size=5-60 circularity=0.70-1.00 summarize"); // axons bleb  // Save the measurement data as an Excel file  saveAs("Results", outputDir + baseName + "_nfl_rawdata.xls");  // Close all images and reset ROI Manager  close(baseName + "_nfl_rawdata.xls");  run("Close All");  run("Clear Results");  roiManager("Reset");  } |

**Western blot**

Cells were lysed using RIPA buffer with cOmplete™ Mini Protease Inhibitor Cocktail (Roche) and centrifuged at 36,000g for 30 minutes at 4°C to obtain whole cell lysates. Pierce™ BCA Protein Assay Kit (Thermo Scientific) was used according to manufacturer’s protocol to determine the protein concentration.

Western blot samples were prepared with 6x Lämmli buffer and heated at 95°C for 5min prior to gel electrophoresis. 10µg/20µl of protein was loaded to each well in 4–15% Mini-PROTEAN® TGX™ Precast Protein Gels (Bio-Rad) along with Chameleon® Duo Pre-stained Protein Ladder (Li-Cor) for size reference. Gel electrophoresis with 1x running buffer was conducted at 100V for 10-15 minutes before increasing to 120V for an additional 55-60 minutes. Protein bands were transferred to PVDF membrane via semi-dry transfer with 1x transfer buffer at 0.09A per membrane for 60 minutes. Membranes were blocked with blocking buffer for 1 hour at room temperature followed by 24-48h incubation with primary antibodies. After primary antibody incubation, membranes were washed three times (5-10 minutes each) with TBS-T (TBS with 0.05% Tween20), followed by a 1-hour incubation with secondary antibodies at room temperature. Lastly, membranes were washed three times with TBS-T and once with TBS before drying and scanning. The antibody list can be found in *Table 3* and buffer compositions in *Table 4*.

**MCF7 media**

MCF7 cells were cultured in DMEM/F-12 media with glutamine (Bio&SELL) with 10% FBS (PAN-Biotech GmbH), 1% MEM Non-Essential Amino Acids (Gibco) and 1% Penicillin-Streptomycin (Gibco).

**Coating of MEA plates**

48-well MEA plates (Axion Biosystems) were coated with 0.07% polyethylenimin (Sigma) in 1× borate buffer (ThermoScientific). On top of the recording electrodes (avoiding the reference electrodes), a 7-9µl drop of the above coating solution was added to the centre of each well. After incubation for 1 hour at 37°C, the wells were rinsed 4 times with sterile distilled water 400µl/well and dried overnight under a cell culture hood before 7-9µl Geltrex (Gibco) droplets were added to each well and subsequently dried for 1 hour under a cell culture hood.

**Live Cell Calcium Imaging**

iPSC-DSN were seeded onto 8-chamber slides (ibidi) at 100 000 cells/well. Before imaging, cells were loaded with Fluo-4 AM (Invitrogen) reconstituted in 0.02% Pluronic™ F-127(Life Technologies) and diluted in a standard HEPES buffered solution (130mM NaCl, 4.7mM KCl, 1mM MgSO4, 1.2mM KH2PO4, 1.3mM CaCl2, 20mM HEPES and 5mM glucose, pH7.4). After incubation for 30 minutes at 37°C, cells were imaged with Leica TCS SP II with a Leica DFC3000G camera. Chemical stimulation was conducted with 100µM ATP (Tocris), 10µM icilin (Tocris) or 10µM capsaicin (Hello Bio). Fluorescence signals were recorded at 1 Hz and positive responses were defined as at least 20% increase of baseline.

**Blinding**

To minimize bias, the researcher was blinded to the clinical CIPN status of the patient donors when receiving the iPSCs and performing experiments for this study. Blind analysis was conducted, wherein treatment conditions of the experiments were not revealed, by data analysts when assessing the outcome.

**Sample Size Justification**

Sample sizes were estimated based on prior works from our lab (Schinke et al., 2021) and agreed on with the Core Unit pluripotent Stem Cells and Organoids as well as the Core Unit Bioinformatics at the Berlin Institute of Health.

## ***Table 1***. Differentiation timeline.

| **Day** | **Media** | | **Supplements** |
| --- | --- | --- | --- |
| -2 | Seed at 200,000-400,000 cells/well in maintenance media | | Y-27632 |
| -1 | Media change into maintenance media | |  |
| 0 | KSR medium | | LDN-193189 + SB-431542 |
| 1 |  |  |  |
| 2 |  |  | LDN-193189 + SB-431542  + CHIR99021 + DAPT + SU5402 |
| 3 |  |  |  |
| 4 | 75% KSR medium | 25% N2B27 medium |  |
| 5 |  |  |  |
| 6 | 50% KSR medium | 50% N2B27 medium | CHIR99021 + DAPT + SU5402 |
| 7 |  |  |  |
| 8 | 25% KSR medium | 75% N2B27 medium |  |
| 9 |  |  |  |
| 10 | N2B27 medium | |  |
| 11 | Reseed cells in N2B27 media | | βNGF, BDNF, NT3, GDNF |

## ***Table 2*.** Summary of Selected Experimental Timepoints.

| **Experiment** | **Figure** | **Drug** | **Timepoint** | **Reason for Selected Timepoint** |
| --- | --- | --- | --- | --- |
| Immuno-fluorescence (IF) images | 1 | All 4 drugs | 72 hours | Latest timepoint that shows prominent increase in c-Jun expression and axonal blebbing in iPSC-DSN when treated with any of the four drugs without SP600125 |
| Western blot | 2 | PTX, BTZ, CDDP | 48 hours | c-Jun and p-c-Jun protein levels increase at 48h upon treatment with PTX, BTZ or CDDP, and as early as 24h post-treatment with VCR |
|  |  | VCR | 24 hours |  |
| Viability assay | 3 | PTX | 72 hours  (24h & 48h in Supplemental Figure S4) | iPSC-DSN treated with PTX alone only started to decrease in viability at 72h while VCR declined from 24h (but variance in distribution was great) and BTZ / CDDP declined from 48h. Ultimately, 48h was selected for comparison across VCR, BTZ and CDDP while 72h was selected for PTX. |
|  |  | VCR, BTZ, CDDP | 48 hours  (24h & 72h in Supplemental Figure S4) |  |
| IF images | 3 | PTX | 72 hours | To match the selected timepoints for viability assay |
|  |  | VCR, BTZ, CDDP | 48 hours |  |
| MEA | 4 | All 4 drugs | 24 hours  (48h & 72h in Supplemental Figure S5) | Monitored electrical activity up to 72h, but concerned about decline in viability and hence decline in functionality in timepoints beyond 24h |
| RNAseq | 5 | PTX | 48 hours | In expectance of RNA levels to rise earlier than protein levels, we selected 16h for VCR, BTZ and CDDP for transcriptomic studies. Selected timepoint for PTX remained at 48h because viability of PTX-treated iPSC-DSN only saw decline at 72h. |
|  |  | VCR, BTZ, CDDP | 16 hours |  |

## ***Table 3.*** List of antibodies used for immunofluorescence and Western blot.

| **Antibody** | **Company** | **Catalogue** | **Dilution** | |
| --- | --- | --- | --- | --- |
|  |  |  | **IF** | **WB** |
| **Primary** | | | | |
| Mouse anti-Neurofilament light chain | Uman Diagnostics | UD2 | 1:1 000 | - |
| Rabbit anti-c-Jun | Cell Signalling | #9165 | 1:400 | 1:1 000 |
| Rabbit anti-Phospho-c-Jun (Ser73) | Cell Signalling | #9164 | - | 1:1 000 |
| Mouse anti-GAPDH | proteintech | 60004-1-Ig | - | 1:20 000 |
| Rabbit anti-Peripherin | ThermoFisher | PA1-10018 | 1:1 000 | - |
| Rabbit anti-TRPV4 | LifeSpan | LS-C94498 | 1:100 | - |
| Rabbit anti-Na_V_1.7 (SCN9A) | Alomone | ASC-008 | 1:100 | - |
| Mouse anti-beta III tubulin | R&D systems | MAB1195 | 1:300 | - |
| Rabbit anti-TRPV1 | Sigma | SAB3501027 | 1:100 | - |
| **Secondary** | | | | |
| Goat anti-mouse IgG Alexa Fluor™ 488 | Invitrogen | A-11001 | 1:600 | - |
| Goat anti-Mouse IgG Alexa Fluor™ 568 | Invitrogen | A-11004 | 1:600 | - |
| Goat anti-rabbit IgG Alexa Fluor™ 488 | Invitrogen | A-11008 | 1:600 | - |
| Goat anti-rabbit IgG Alexa Fluor™ 568 | Invitrogen | A-11011 | 1:600 | - |
| Donkey anti-mouse IgG IRDye 680RD | Li-Cor | 926-68072 | - | 1:15 000 |
| Donkey anti-rabbit IgG IRDye 800CW | Li-Cor | 926-32213 | - | 1:15 000 |

## ***Table 4.*** Western blot buffers.

| **Buffer Type** | **Components** | **Amount** |
| --- | --- | --- |
| RIPA buffer | Tris (pH 7.4) | 50mM |
|  | NaCl | 150mM |
|  | Triton X100 | 1% |
|  | Sodium dodecyl sulfate (SDS) | 0.1% |
|  | Sodium deoxycholate | 1% |
| 6× Lämmli buffer | Tris-HCl | 0.5M |
|  | glycerol | 20% |
|  | SDS | 2% |
|  | Bromophenol blue | 0.06% |
|  | β-mercaptoethanol | 0.02% |
| 1× Running buffer | Tris | 25mM |
|  | Glycine | 192mM |
|  | SDS | 0.1% |
|  | Distilled water | 1 litre |
| 1× Transfer buffer | Tris | 25mM |
|  | Glycine | 192mM |
|  | SDS | 0.01% |
|  | Methanol | 100ml |
|  | Distilled water | 900ml |
| Tris-buffer saline (TBS) | Tris-HCl (pH7.4) | 25mM |
|  | NaCl | 150mM |
|  | Distilled water | 1 litre |
| Blocking buffer | TBS | 1:1 |
|  | Intercept® (TBS) Blocking Buffer (Li-Cor) |  |
