# Supplementary material for: c-Jun inhibition mitigates chemotherapy-induced neurotoxicity in iPSC-derived sensory neurons
Source: Cell Death Discov. 2025 Nov 13;11:529. doi: 10.1038/s41420-025-02847-5 (PMC12615653; doi:10.1038/s41420-025-02847-5)
Supplement: Supplementary file 2 — Supplemental Figures with Captions [file 41420_2025_2847_MOESM2_ESM.docx]

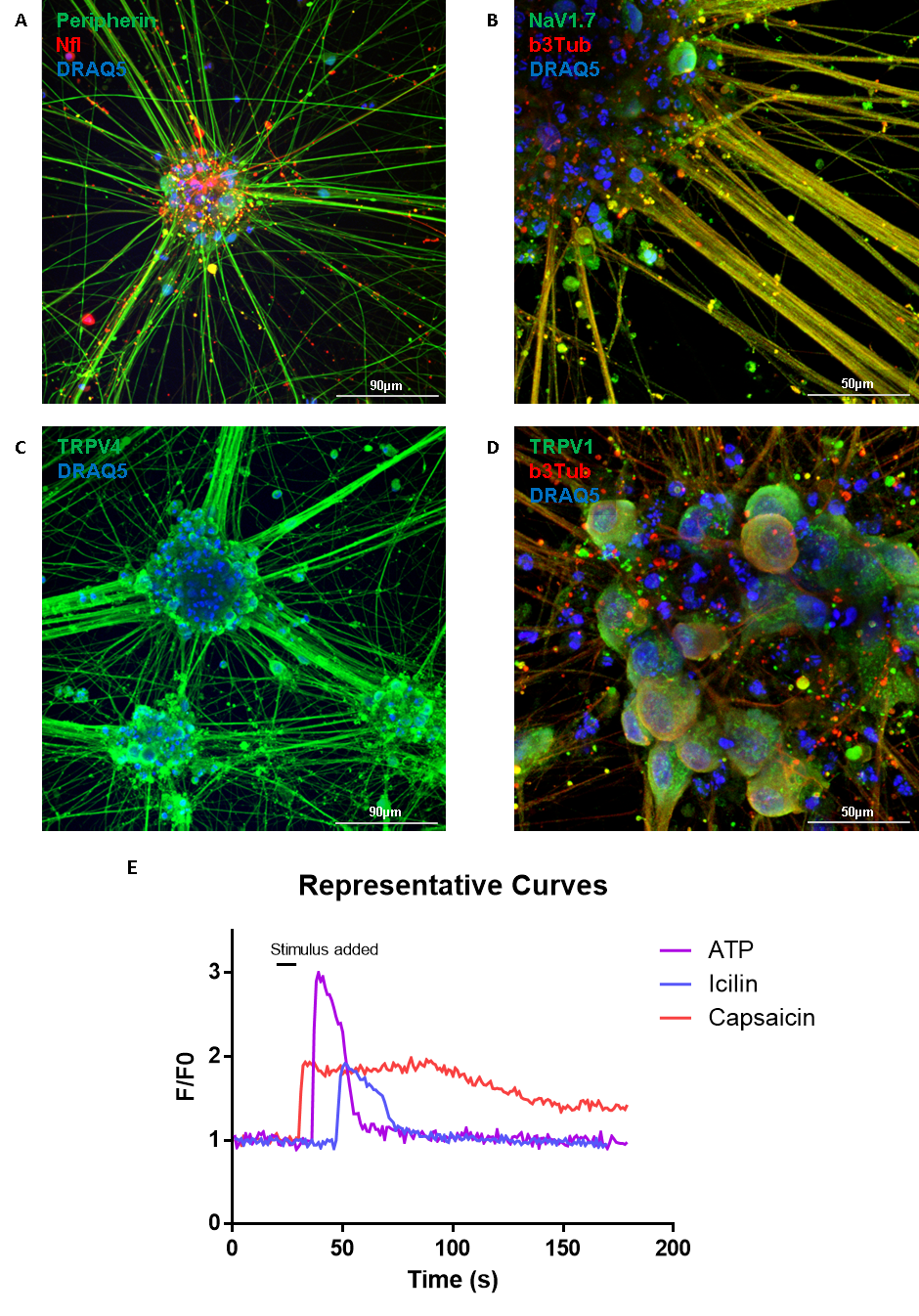


**Supplemental Figure S1. Characterization of iPSC-DSN from BIHi264-A and BIHi265-A patient cell lines.** The iPSC-DSN express sensory neuronal markers of **(A)** peripherin, neurofilament light chain, **(B)** Na_V_1.7, **(C)** TRPV4, and **(D)** TRPV1 ion channels. Live cell calcium imaging shows typical sensory neuron responses to chemical stimuli with 100µM ATP, 10µM icilin and 10µM capsaicin. **(E)** Representative traces of 4 replicates from 2 independent experiments with 2 separate differentiation batches. Response profile to each stimulus is normalized to baseline (F/F0).


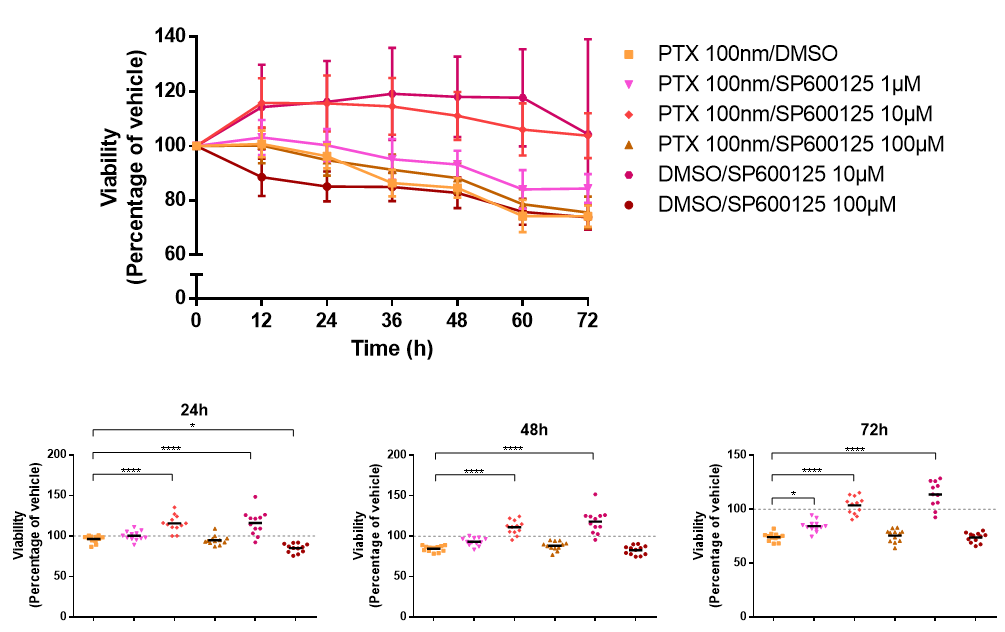


**Supplemental Figure S2. SP600125 improves viability in paclitaxel-treated alternate patient iPSC-DSN cell line.** Live viability test conducted on BIHi265-A patient cell line showed decreasing cell viability upon treatment with 100nM paclitaxel which could be salvaged upon addition of SP600125. Replicates from 2 plates with 6 wells per condition was factored into statistical analysis using one-way ANOVA to check for statistical significance. *p < 0.05, **p < 0.01, ***p < 0.001, ****p < 0.0001


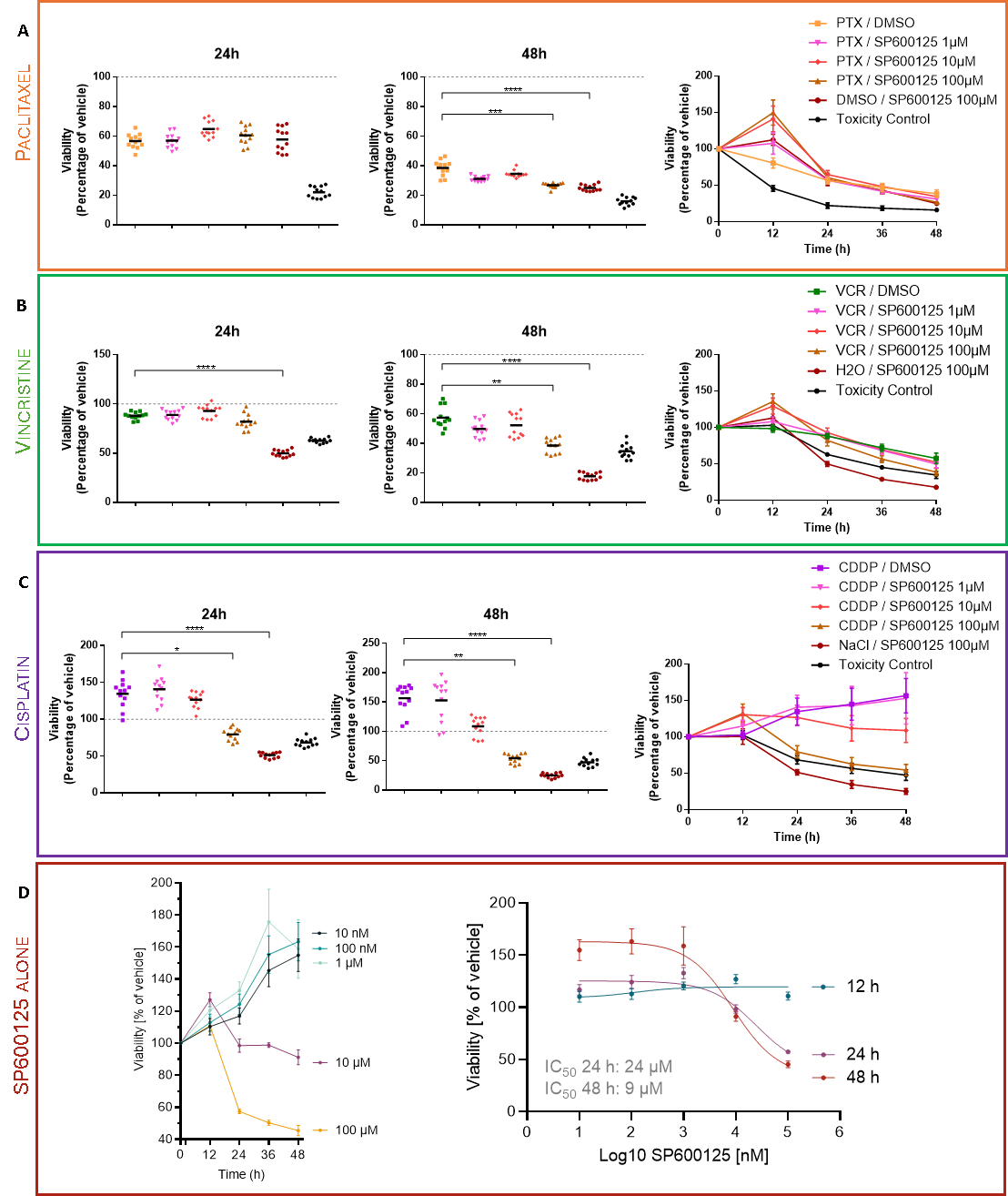


**Supplemental Figure S3. MCF7 breast cancer cells incubated with chemotherapy drugs and SP600125 over time.** Live viability test was conducted on MCF7 cells to investigate the effect of **(A)** 100nM paclitaxel, **(B)** 100nM vincristine, **(C)** 10µM cisplatin upon co-incubation with SP600125 over a 48-hour period. Incubation with **(D)** SP600125 alone showed that while MCF7 cells continue proliferating over the course of 48h at low concentrations of SP600125, 10µM stopped proliferation and led to a decline in viability in a time-dependent manner. The accompanying dose response curve (normalized to individual well baseline) confirms an IC50 of approximately 9µM at 48h for MCF7 cells. Curves show the mean with 95% confidence interval. Two independent experiments were pooled, with 6 technical replicates per condition per experiment (12 replicates in total per condition). Kruskal Wallis test, followed by Dunn’s multiple comparisons test, was used to evaluate statistical significance. *p < 0.05, **p < 0.01, ***p < 0.001, ****p < 0.0001


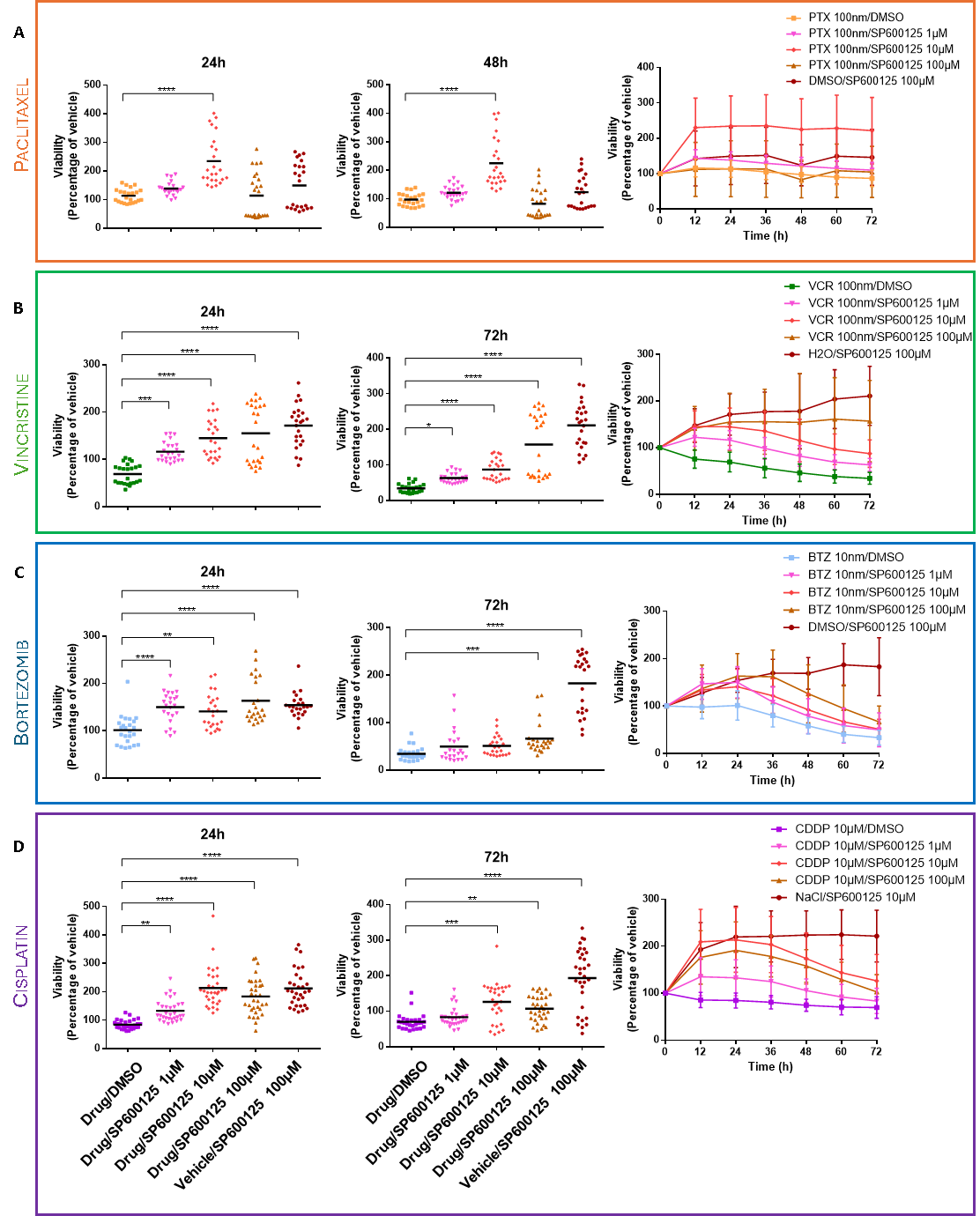


**Supplemental Figure S4. iPSC-DSN cell viability for all timepoints.** Viability readouts of iPSC-DSN treated with **(A)** paclitaxel at 24- and 48-hour timepoints, or **(B)** vincristine, **(C)** bortezomib or **(D)** cisplatin at 24- and 72-hour timepoints in the presence and absence of SP600125 and respective graphs plotted for viability over time. Statistical significance was obtained with the Kruskal Wallis test, followed by Dunn’s multiple comparisons test. *p < 0.05, **p < 0.01, ***p < 0.001, ****p < 0.0001


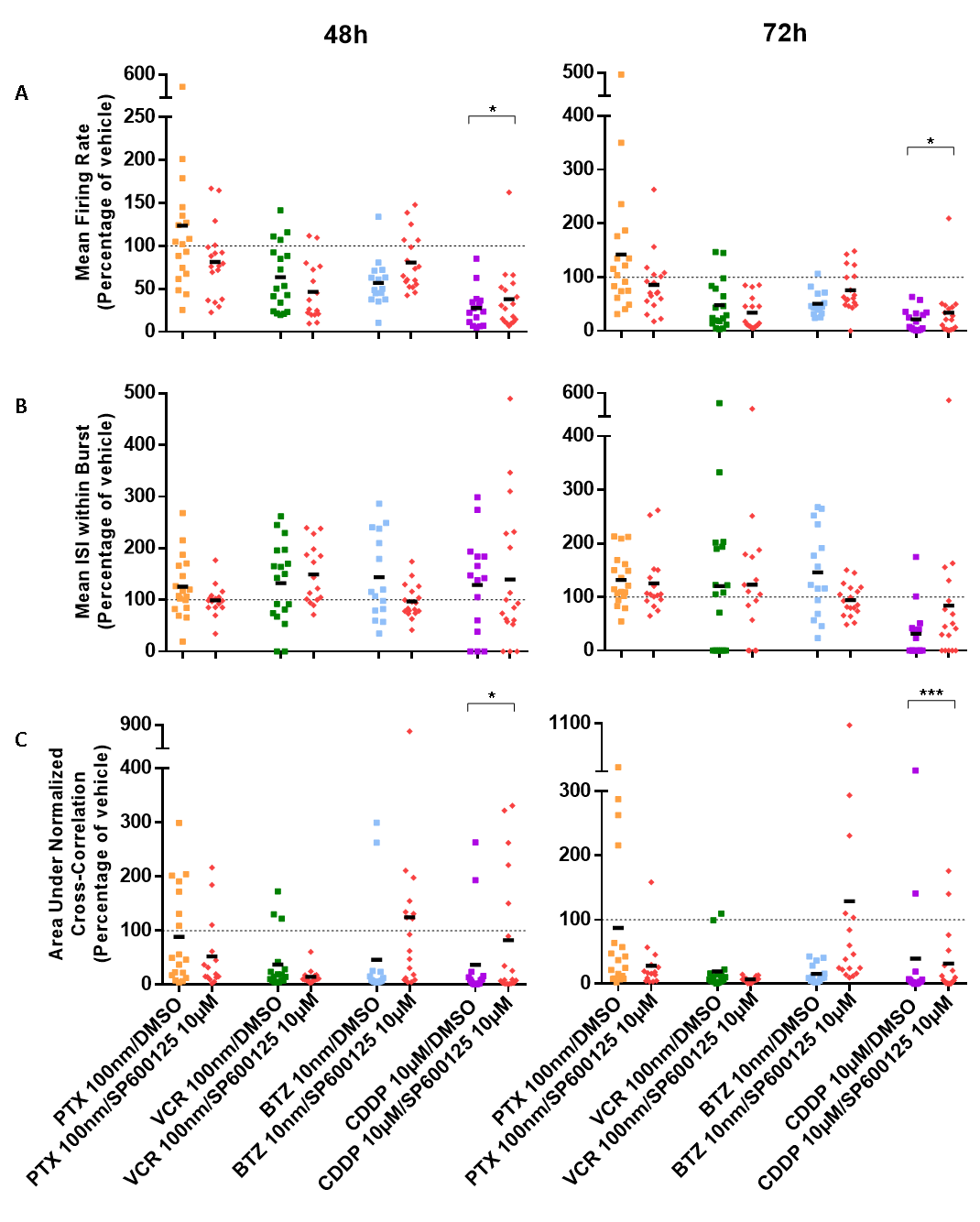


**Supplementary Figure S5. Electrophysiological response of iPSC-DSN treated with cytotoxic drugs in the presence of SP600125 at 48- and 72-hour timepoints.** Dot plots display **(A)** Mean Firing Rate (MFR), **(B)** Mean Interspike Interval (ISI) within a burst and **(C)** Area Under Normalized Cross-correlation as a measure of synchronicity. Dotted line represents vehicle/DMSO = 100%. Data from 3 plates, with 6 wells per treatment condition for combined total of n = 18 per group, was statistically evaluated with the Mann-Whitney U test (two-tailed). **p < 0.01


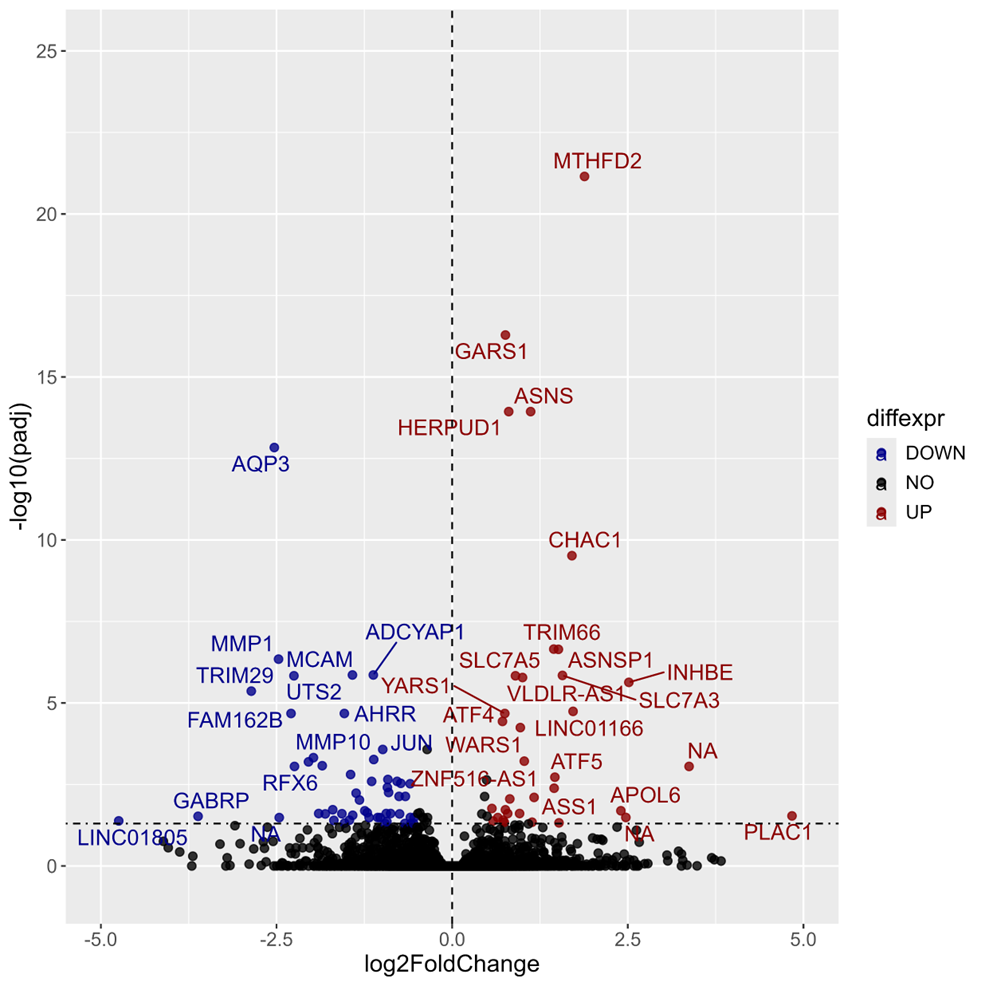


**Supplemental Figure S6. Differential RNA expression in iPSC-DSN after 48h incubation with 10µM SP600125 or vehicle.** JNK inhibition by 10µM SP600125 led to the expected downregulation of JUN as a direct phosphorylation target of JNK. The differential expression of genes of extracellular remodelling (MMP1, MMP10), amino acid transport and metabolism (e.g., SLC7A5, ASNSP1, MTHFD2) and the integrated stress response (ATF4, ATF5) suggest some downstream and potential off-target effects of SP600125 that may, in part, contribute to the observed endpoints.


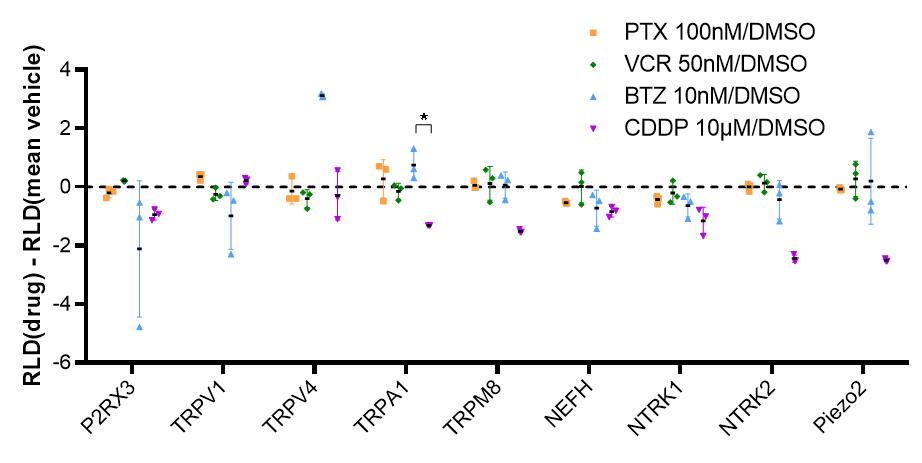


**Supplemental Figure S7. mRNA levels of Aβ, Aδ and C fibre markers expressed within iPSC-DSN cell culture population.** iPSC-DSN express markers for Aβ, Aδ and C fibres, such as P2RX3, TRPV1, TRPV4, TRPA1, TRPM8, NEFH (NF200), NTRK1 (TRKA), NTRK2 (TRKB) and Piezo2. Comparison across four different drug treatments show slight differences in iPSC-DSN. The statistical significance was calculated using the Kruskal Wallis test, followed by Dunn’s multiple comparisons test. *p < 0.05


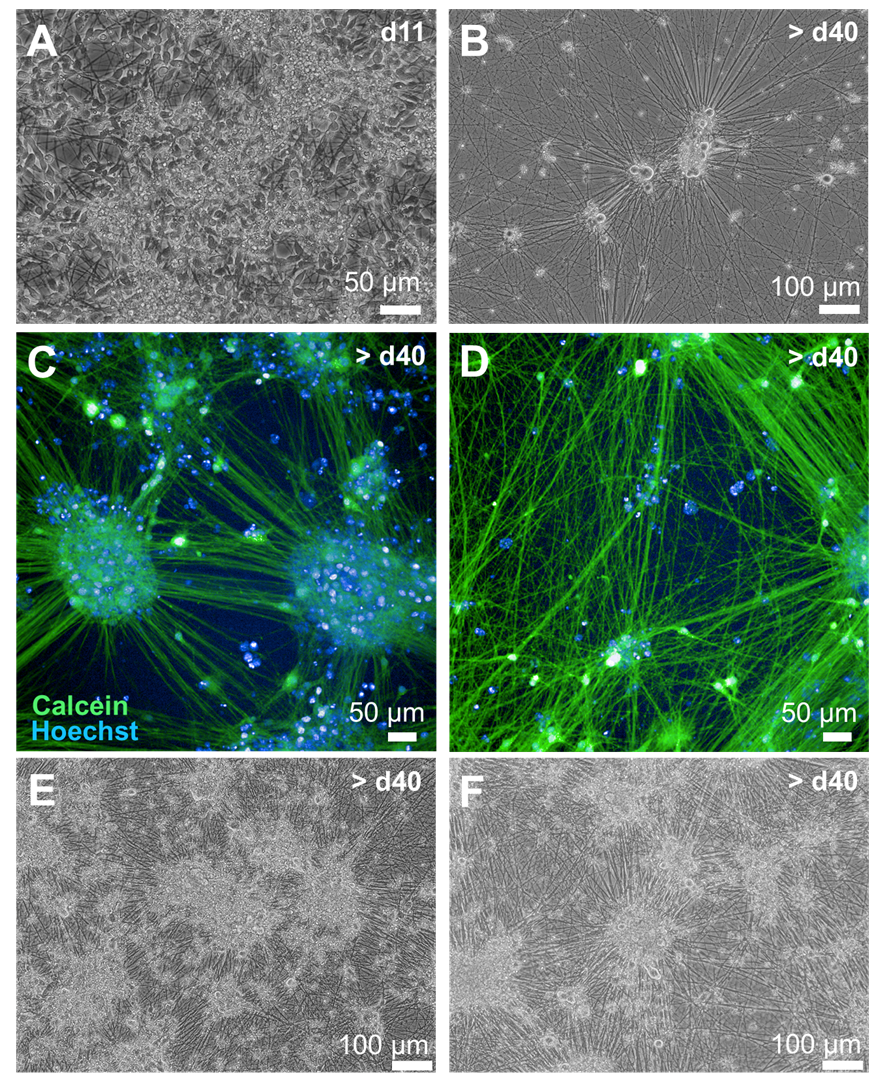


**Supplemental Figure S8. Morphology of iPSC-DSN derived from BIHi264-A (A-D) and BIHi265-A (E-F).** After day 11 **(A)**, iPSC-DSN were reseeded and treated with mitomycin C on day 14 to eliminate proliferating non-neuronal cells. On day 15, iPSC-DSN express on average >99% beta III tubulin and >88% peripherin (Huehnchen et al., 2022). At the time of experiments (>d40), highly pure neuronal cultures were observed in brightfield imaging **(B, E, F)** and live cell calcein imaging **(C-D)**.


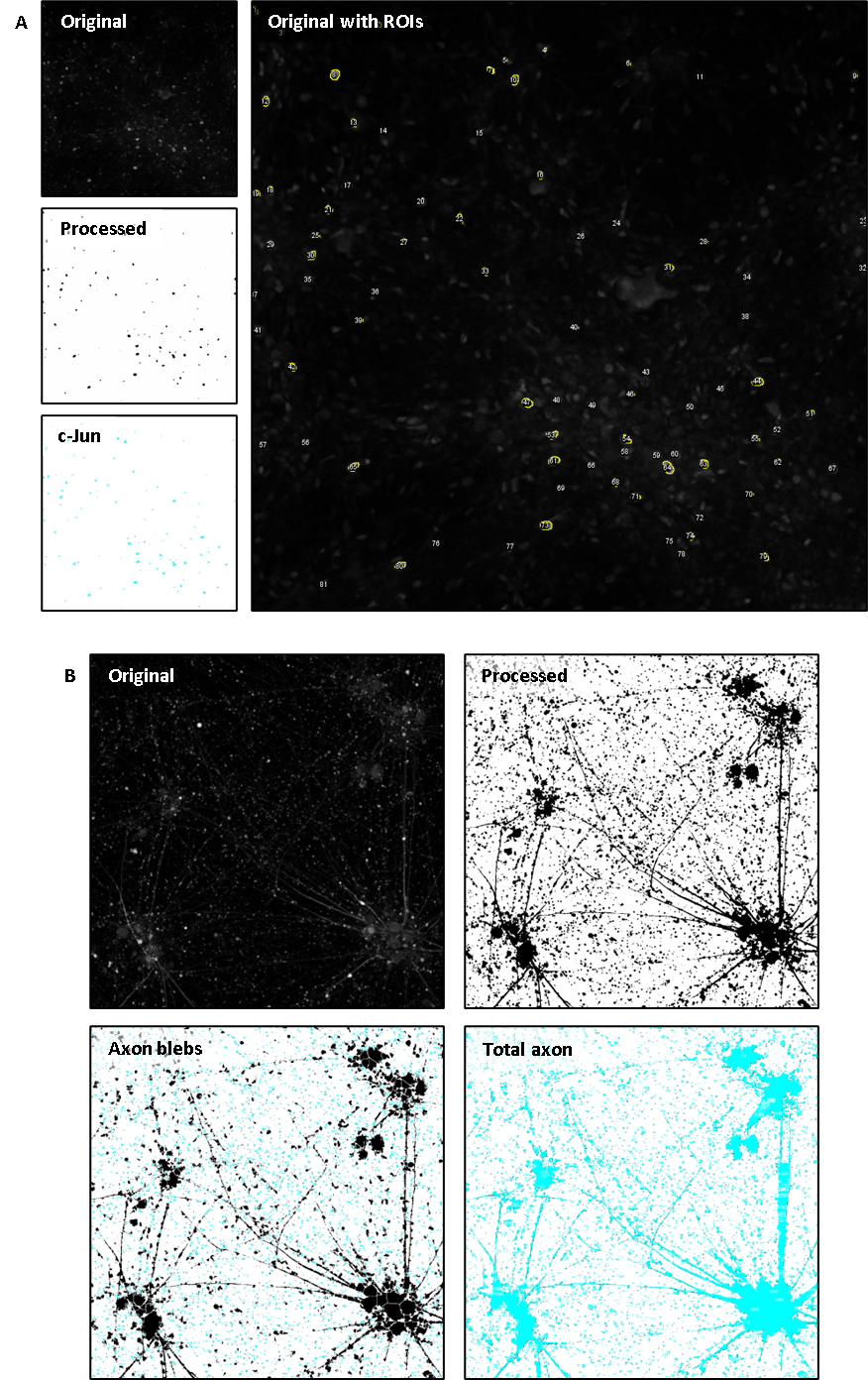


**Supplemental Figure S9. Analysis of HCS Images with ImageJ.** Original HCS images were processed via 8-bit conversion and threshold adjustments. Particle analysis was then applied to obtain regions of interest. **(A)** For quantification of c-Jun in IF images, these ROIs were then applied to the original image to measure fluorescent intensity of immunostaining. The quantification of DRAQ5 in IF images was conducted in the same manner. **(B)** For axonal bleb quantification, ROIs for axon blebs and ROIs for total axon area were used.


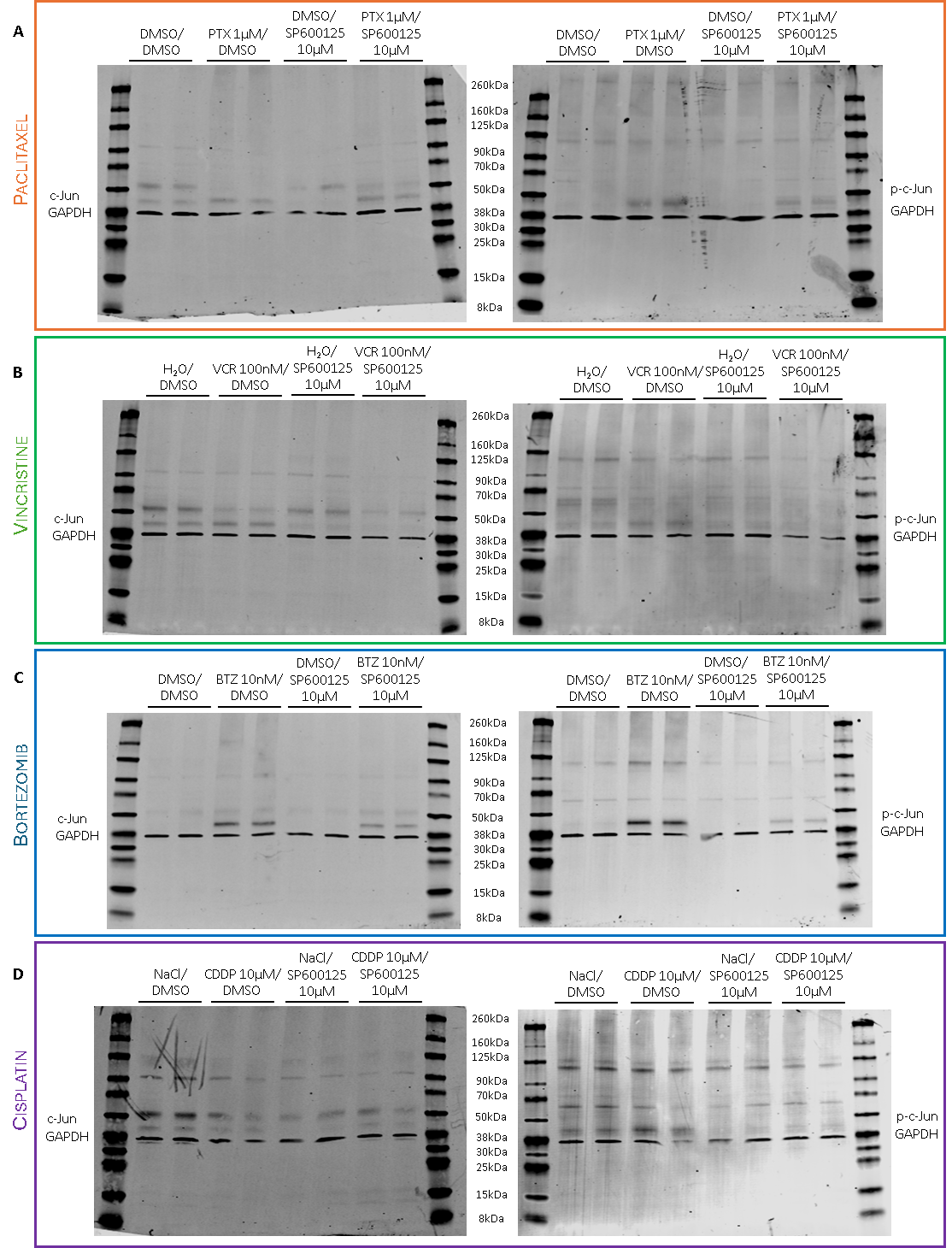


**Supplemental Figure S10. Uncropped Western blot scans.** Whole cell lysates from iPSC-DSN treated with **(A)** paclitaxel, **(B)** vincristine, **(C)** bortezomib or **(D)** cisplatin in the presence and absence of SP600125 were blotted for c-Jun and phosphorylated c-Jun.


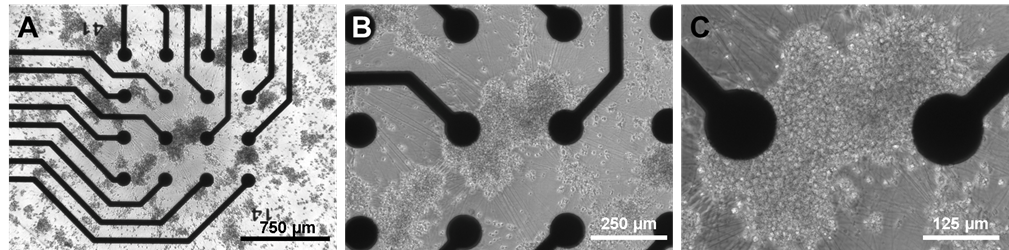


**Supplemental Figure S11. Representative images of iPSC-DSN cultured on MEA plates.** iPSC-DSN were grown on 48-well MEA plates with 16 electrodes per well until experimented were conducted after 50 days.


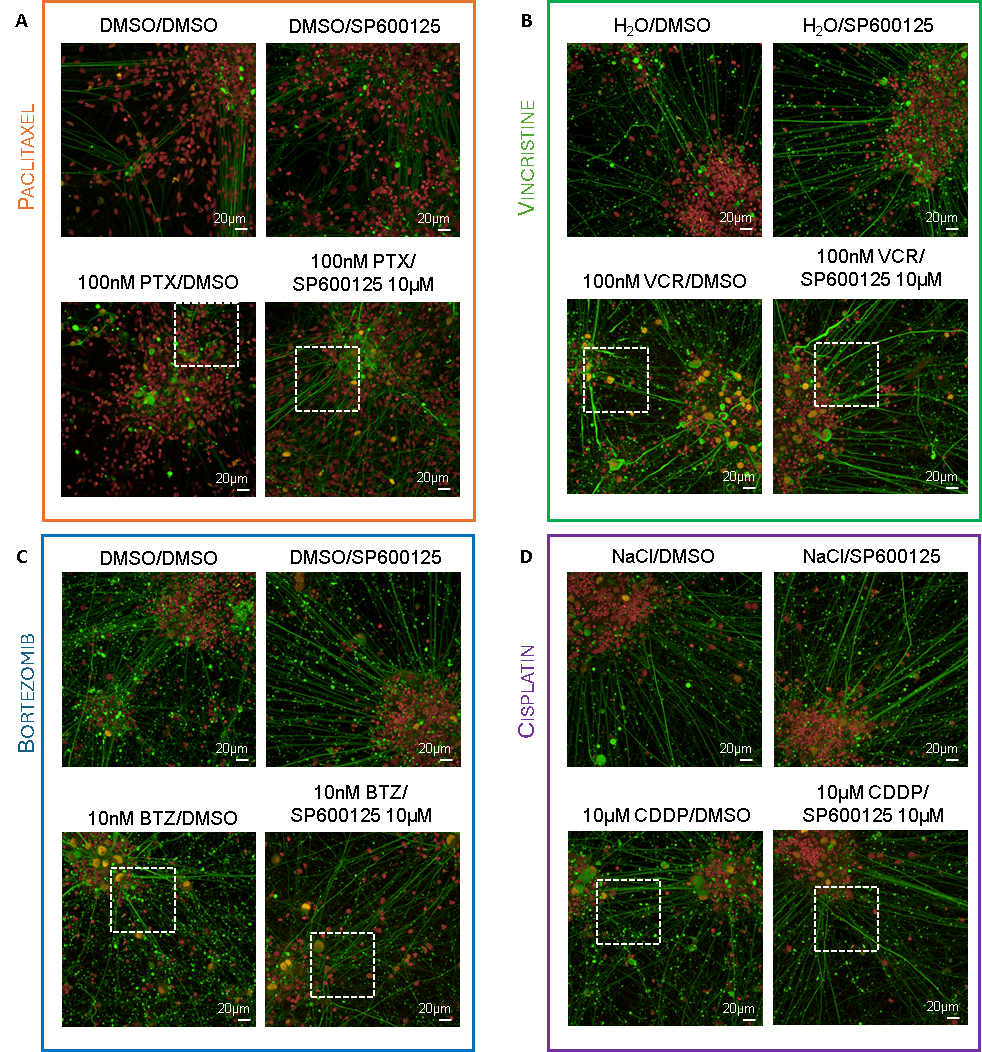


**Supplemental Figure S12. Morphology of iPSC-DSN upon c-Jun inhibition.** iPSC-DSN were treated with **(A)** paclitaxel, **(B)** vincristine, **(C)** bortezomib or **(D)** cisplatin. Confocal images show neurofilament light chain in green, c-Jun in yellow and DRAQ5 in red (scale bar: 20µm). When compared to corresponding vehicle controls, c-Jun expression and axon blebbing increased for all four drugs while the addition of SP600125 improved axon blebbing for PTX- or BTZ-treated iPSC-DSN. Incubation with vehicle control or 10µM SP600125 in the absence of chemotherapeutic agent does not increase c-Jun expression nor axon degeneration. The regions marked by white dotted lines were cropped and enlarged for Figure 3.
