# Supplementary figures and images for: c-Jun inhibition mitigates chemotherapy-induced neurotoxicity in iPSC-derived sensory neurons
Source: Cell Death Discov. 2025 Nov 13;11:529. doi: 10.1038/s41420-025-02847-5 (PMC12615653; doi:10.1038/s41420-025-02847-5)

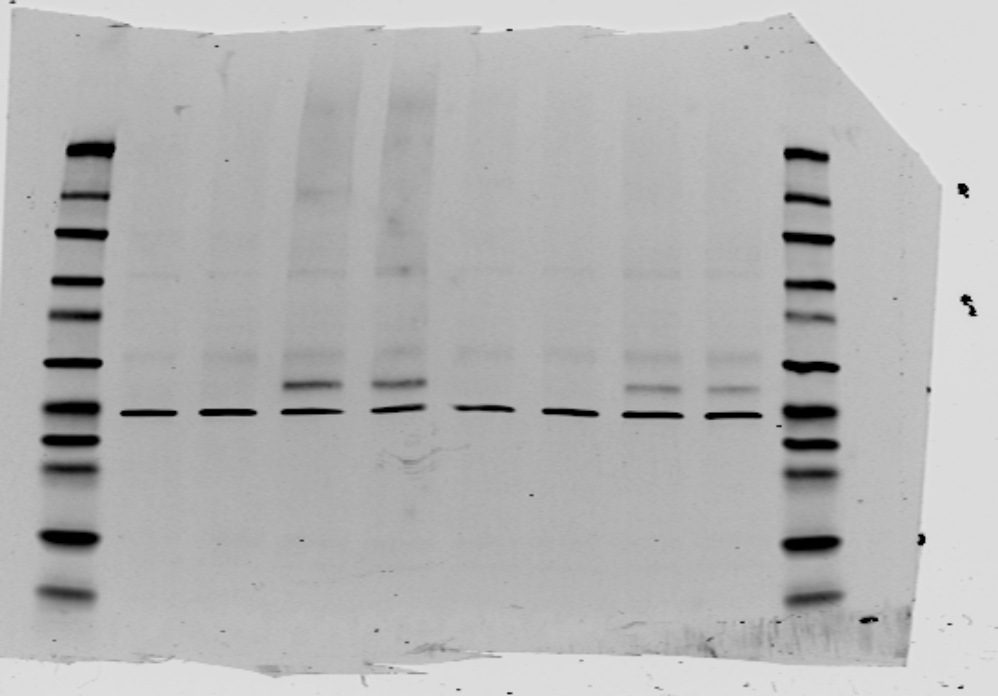

Supplement: Supplementary file 3 — Dataset [file 41420_2025_2847_MOESM3_ESM.zip › Supplemental Files (to be zipped)/(Figure 2 and S10) Western blot/20240627 264a BTZ SP bw c-Jun GAPDH.tif]

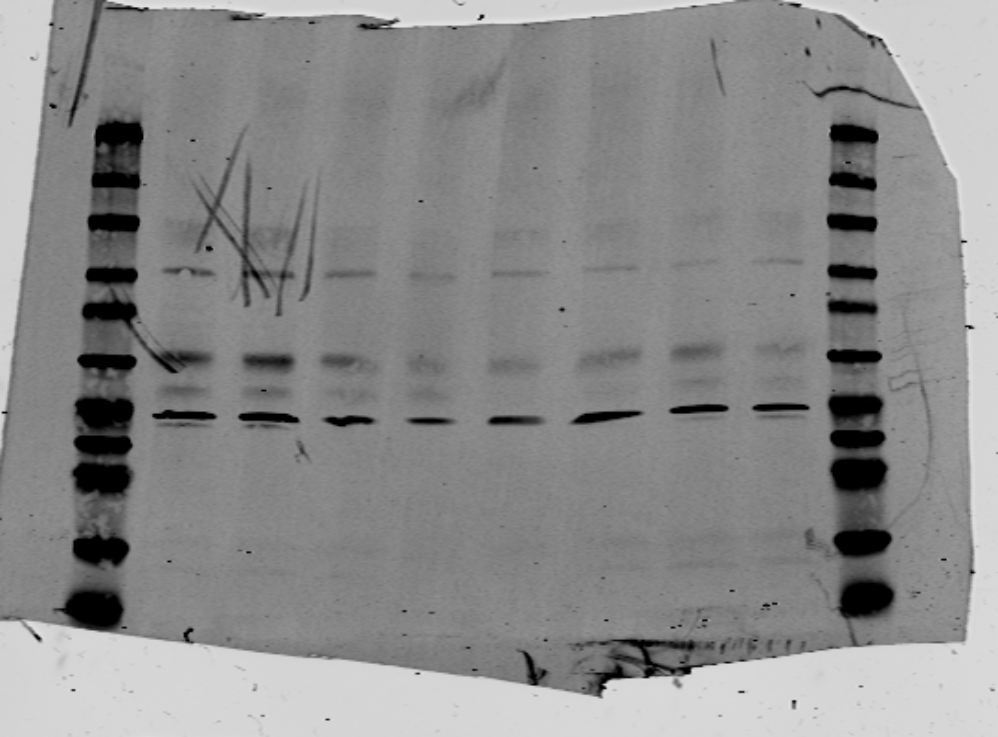

Supplement: Supplementary file 3 — Dataset [file 41420_2025_2847_MOESM3_ESM.zip › Supplemental Files (to be zipped)/(Figure 2 and S10) Western blot/20240726 264a CDDP SP bw c-Jun GAPDH.tif]

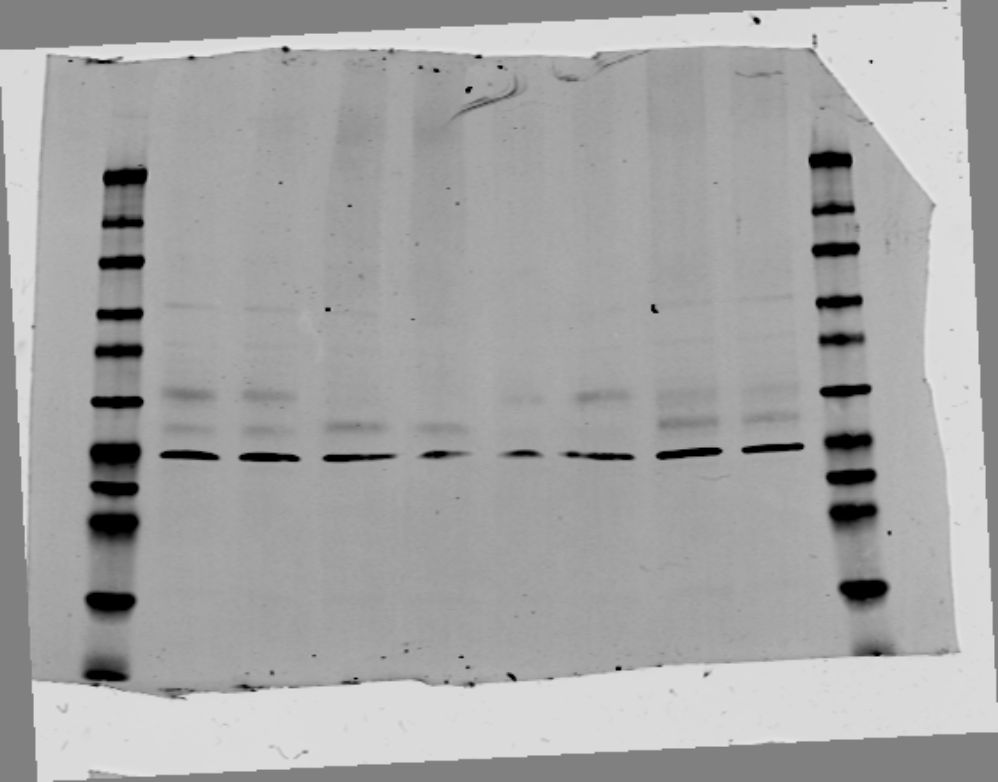

Supplement: Supplementary file 3 — Dataset [file 41420_2025_2847_MOESM3_ESM.zip › Supplemental Files (to be zipped)/(Figure 2 and S10) Western blot/20240726 264a PTX SP bw c-Jun GAPDH.tif]

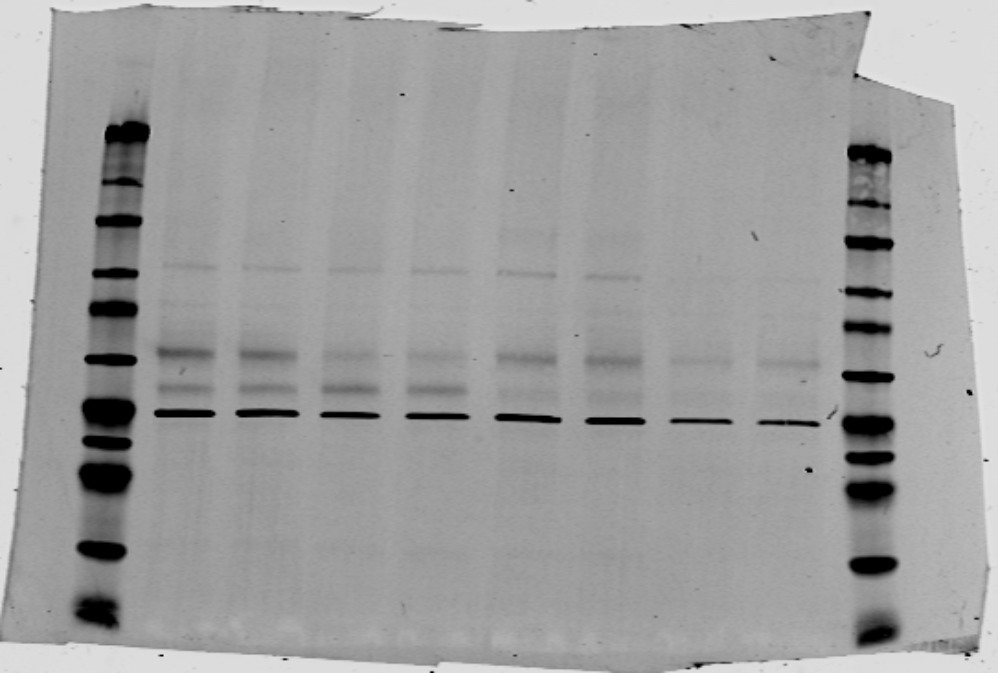

Supplement: Supplementary file 3 — Dataset [file 41420_2025_2847_MOESM3_ESM.zip › Supplemental Files (to be zipped)/(Figure 2 and S10) Western blot/20240726 264a VCR SP bw c-Jun GAPDH.tif]

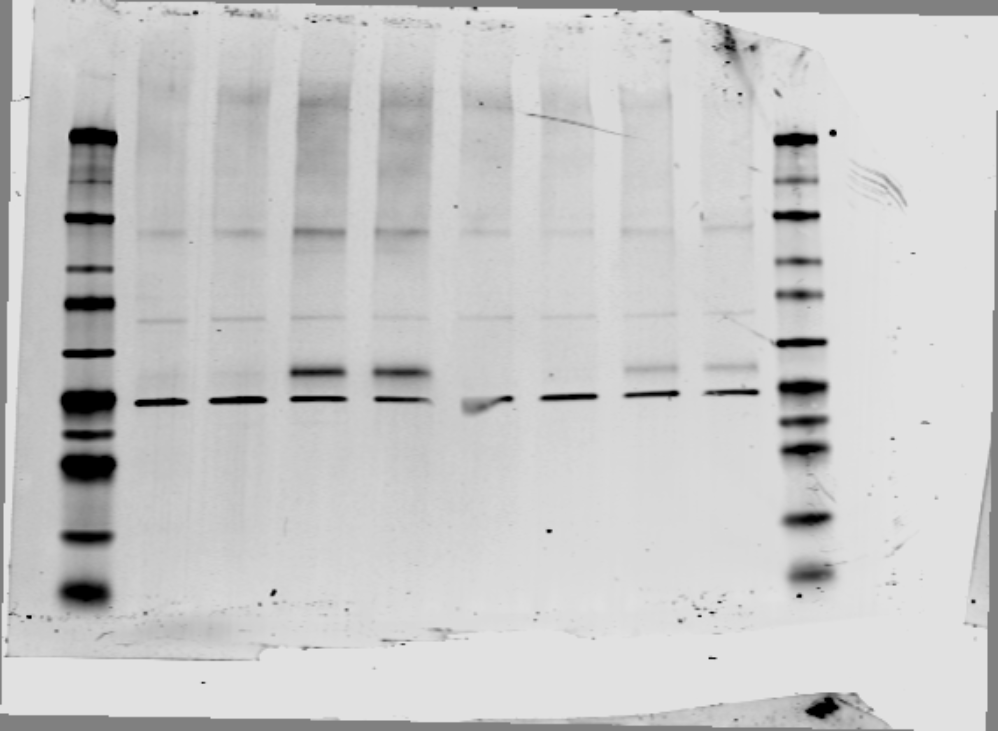

Supplement: Supplementary file 3 — Dataset [file 41420_2025_2847_MOESM3_ESM.zip › Supplemental Files (to be zipped)/(Figure 2 and S10) Western blot/20240814 264a BTZ SP bw p-cJun GAPDH.tif]

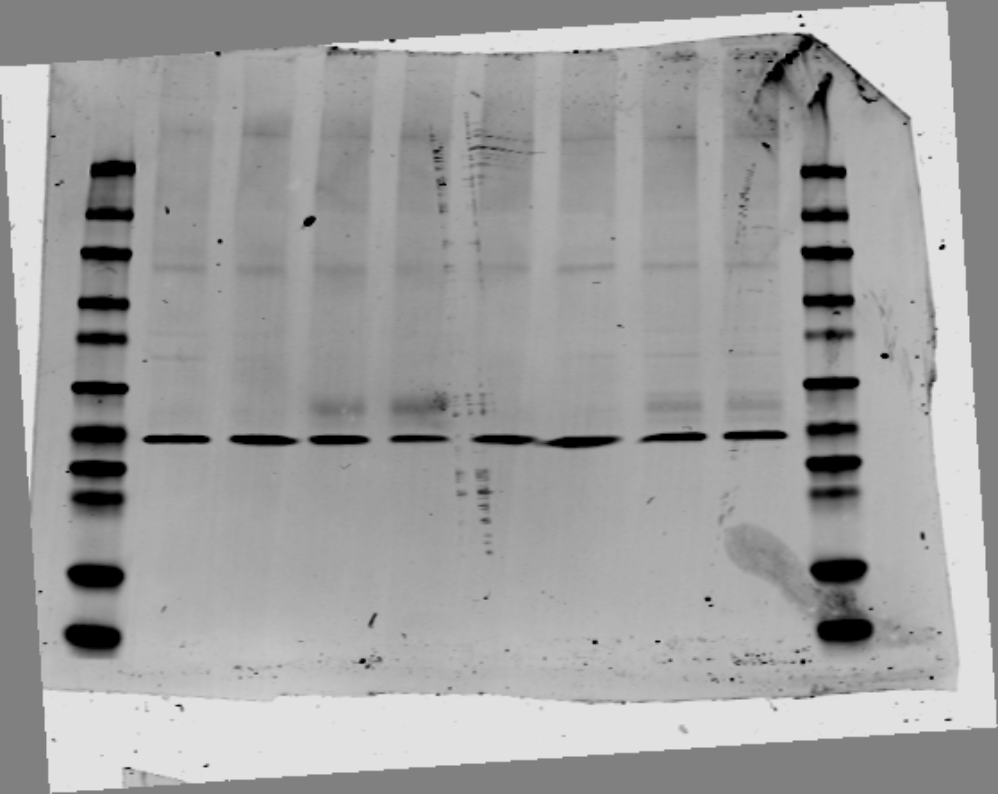

Supplement: Supplementary file 3 — Dataset [file 41420_2025_2847_MOESM3_ESM.zip › Supplemental Files (to be zipped)/(Figure 2 and S10) Western blot/20240814 264a PTX SP bw p-cJun GAPDH.tif]

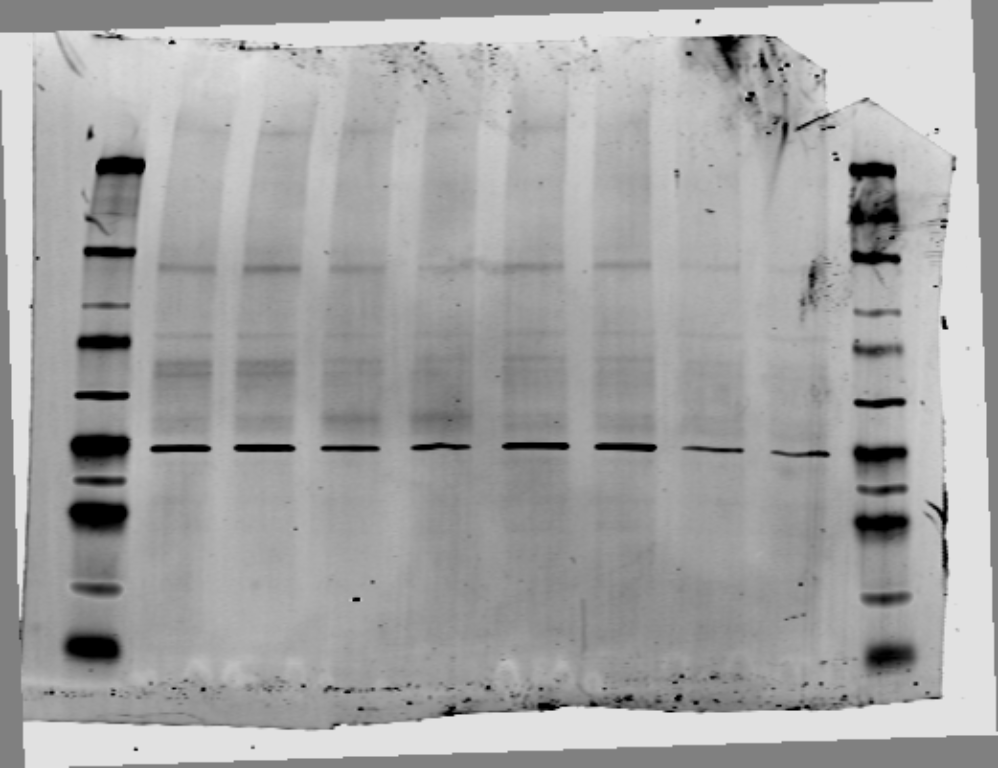

Supplement: Supplementary file 3 — Dataset [file 41420_2025_2847_MOESM3_ESM.zip › Supplemental Files (to be zipped)/(Figure 2 and S10) Western blot/20240814 264a VCR100nm SP bw p-cJun GAPDH.tif]

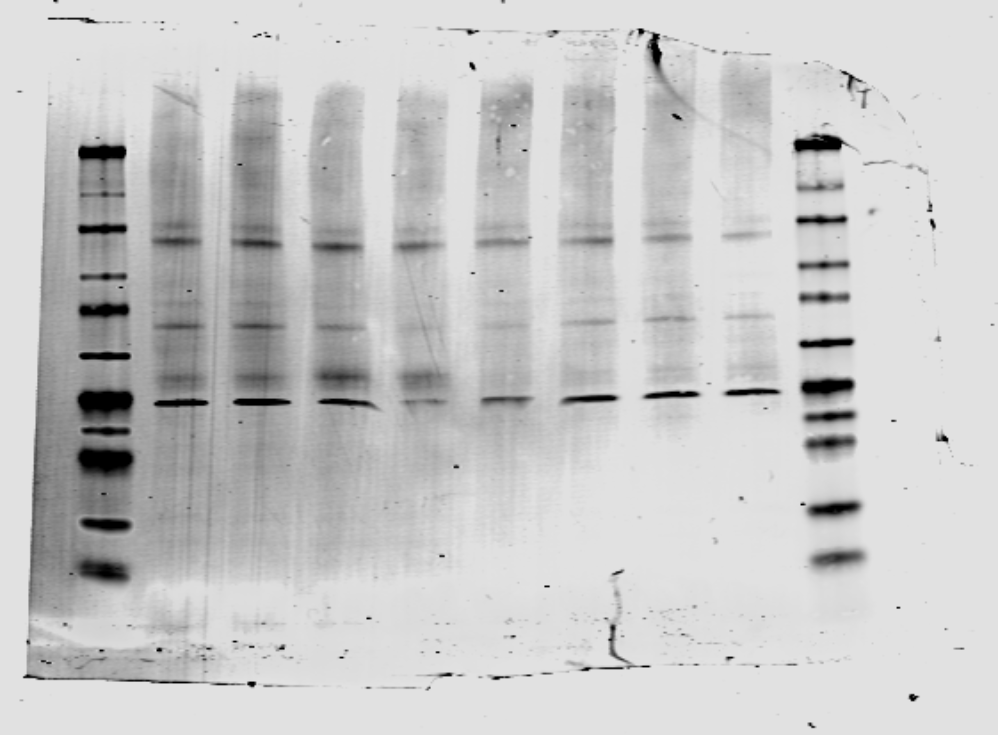

Supplement: Supplementary file 3 — Dataset [file 41420_2025_2847_MOESM3_ESM.zip › Supplemental Files (to be zipped)/(Figure 2 and S10) Western blot/20240826 264a CDDP SP bw p-cJun GAPDH.tif]

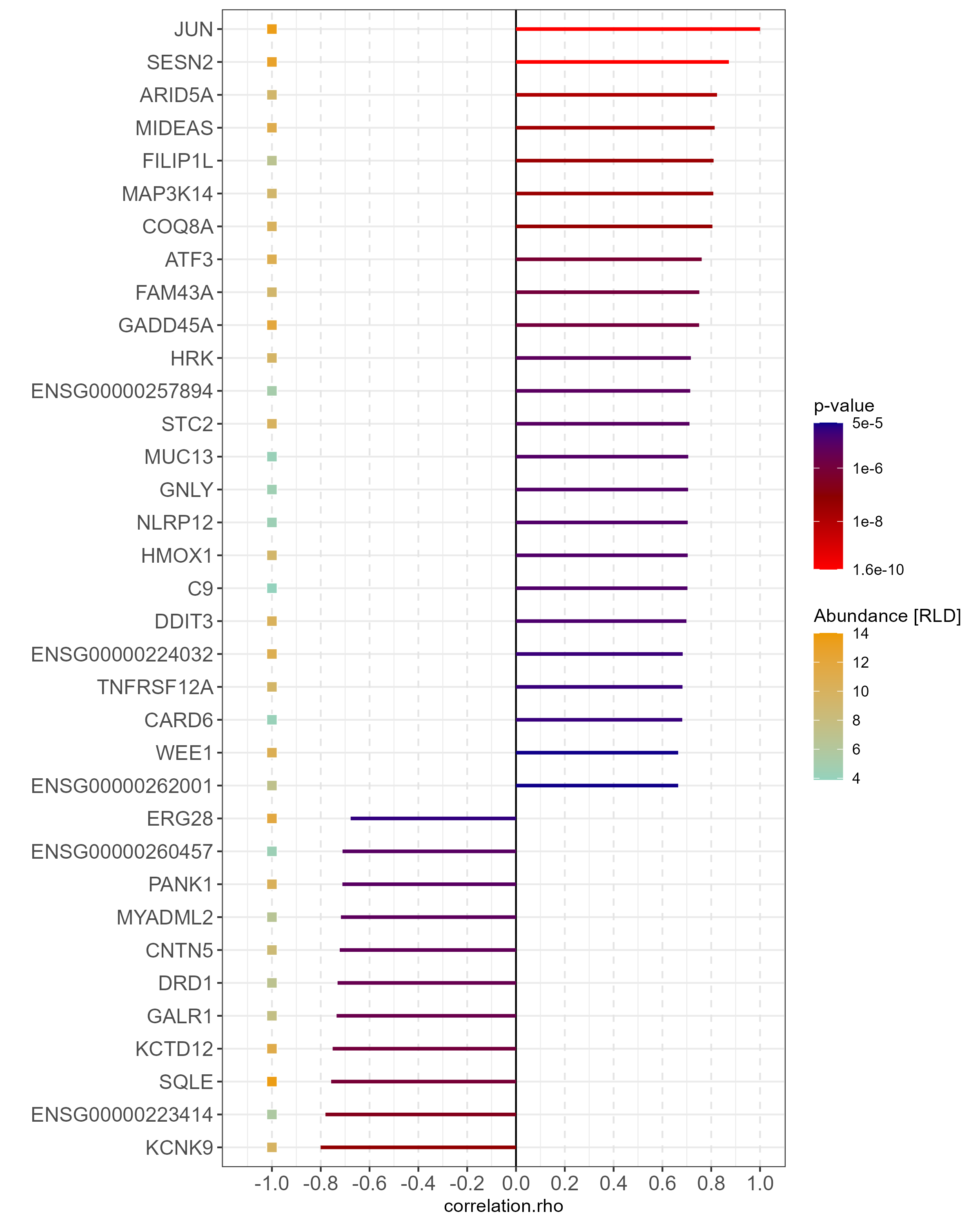

Supplement: Supplementary file 3 — Dataset [file 41420_2025_2847_MOESM3_ESM.zip › Supplemental Files (to be zipped)/(Figure 5 and S6) RNAseq/JUN_Genes_of_interest_plot_with_FLD.tiff]

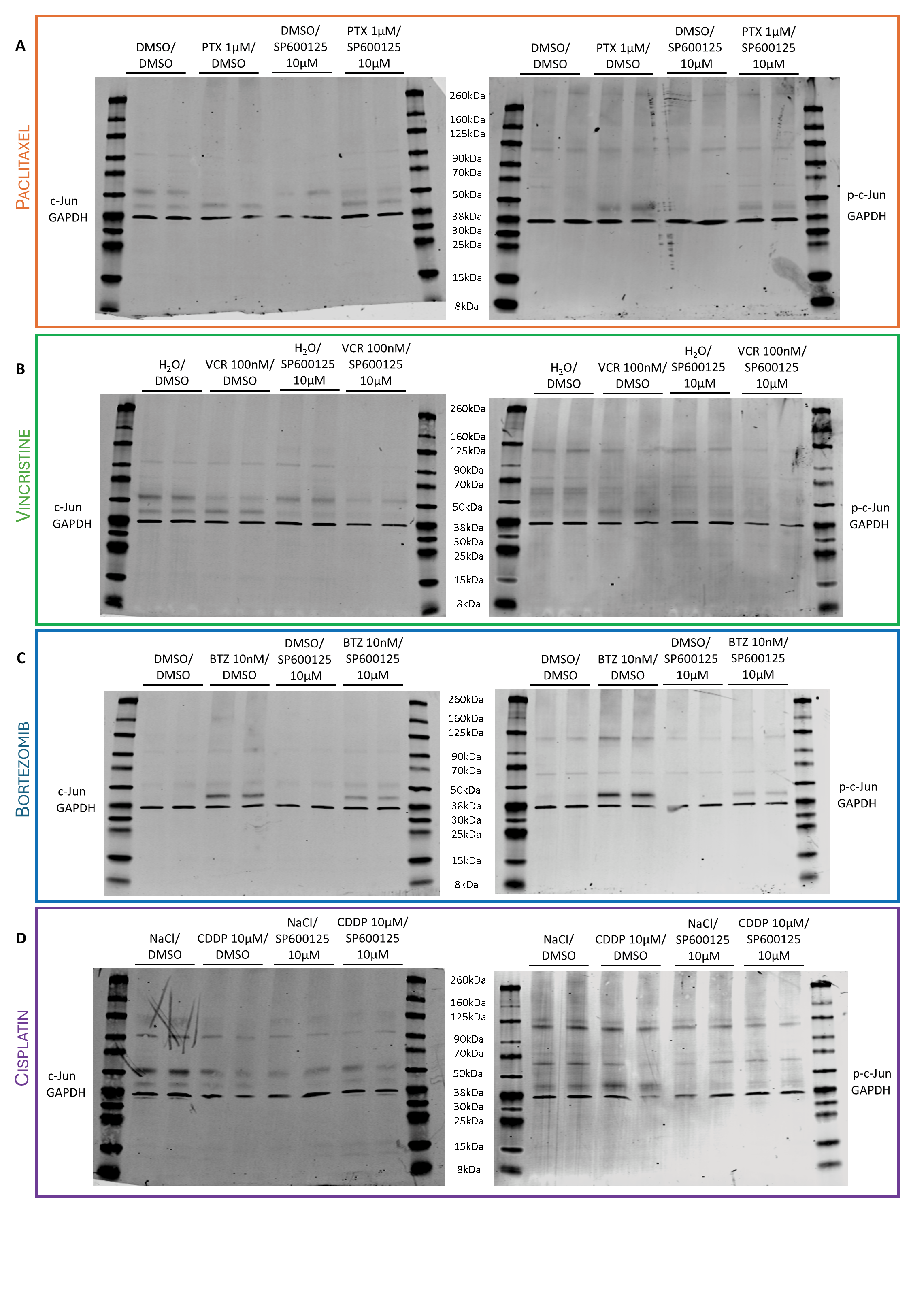

Supplement: Supplementary file 5 — Original Data (of Western blots) [file 41420_2025_2847_MOESM5_ESM.tif]
